# *NbALD1* mediates resistance to turnip mosaic virus by regulating the accumulation of salicylic acid and the ethylene pathway in *Nicotiana benthamiana*

**DOI:** 10.1111/mpp.12808

**Published:** 2019-04-23

**Authors:** Shu Wang, Kelei Han, Jiejun Peng, Jinping Zhao, Liangliang Jiang, Yuwen Lu, Hongying Zheng, Lin Lin, Jianping Chen, Fei Yan

**Affiliations:** ^1^ College of Agriculture and Biotechnology Zhejiang University Hangzhou 310058 China; ^2^ The State Key Laboratory Breeding Base for Sustainable Control of Pest and Disease, Key Laboratory of Biotechnology in Plant Protection of MOA of China and Zhejiang Province, Institute of Virology and Biotechnology Zhejiang Academy of Agricultural Sciences Hangzhou 310021 China; ^3^ Institute of Plant Virology Ningbo University Ningbo 315211 China; ^4^ College of Plant Protection Nanjing Agricultural University Nanjing 210095 China

**Keywords:** ALD1, ethylene, pipecolic acid, resistance, salicylic acid, turnip mosaic virus

## Abstract

AGD2‐LIKE DEFENCE RESPONSE PROTEIN 1 (ALD1) triggers plant defence against bacterial and fungal pathogens by regulating the salicylic acid (SA) pathway and an unknown SA‐independent pathway. We now show that *Nicotiana benthamiana ALD1* is involved in defence against a virus and that the ethylene pathway also participates in *ALD1*‐mediated resistance. *NbALD1* was up‐regulated in plants infected with turnip mosaic virus (TuMV). Silencing of *NbALD1* facilitated TuMV infection, while overexpression of *NbALD1* or exogenous application of pipecolic acid (Pip), the downstream product of *ALD1*, enhanced resistance to TuMV. The SA content was lower in *NbALD1*‐silenced plants and higher where *NbALD1* was overexpressed or following Pip treatments. SA mediated resistance to TuMV and was required for *NbALD1*‐mediated resistance. However, on *NahG* plants (in which SA cannot accumulate), Pip treatment still alleviated susceptibility to TuMV, further demonstrating the presence of an SA‐independent resistance pathway. The ethylene precursor, 1‐aminocyclopropanecarboxylic acid (ACC), accumulated in *NbALD1*‐silenced plants but was reduced in plants overexpressing *NbALD1* or treated with Pip. Silencing of *ACS1*, a key gene in the ethylene pathway, alleviated the susceptibility of *NbALD1*‐silenced plants to TuMV, while exogenous application of ACC compromised the resistance of Pip‐treated or *NbALD1* transgenic plants. The results indicate that *NbALD1* mediates resistance to TuMV by positively regulating the resistant SA pathway and negatively regulating the susceptible ethylene pathway.

## Introduction

Salicylic acid (SA) induces an important plant defence pathway against pathogens by producing pathogenesis‐related proteins (Durrant and Dong, 2004; Fu and Dong, 2013; Gao *et al.*, 2015; Tsuda *et al.*, 2009; Vlot *et al.*, 2009). Research over the last decade has shown that AGD2‐LIKE DEFENCE RESPONSE PROTEIN 1 (ALD1) triggers the basal defence response and systemic acquired resistance (SAR) against bacterial infection in plants by regulating the SA pathway (Cecchini *et al.*, 2015; Song *et al.*, 2004a). Compared with wild‐type plants, *ald1* mutants of *Arabidopsis thaliana* were more susceptible to *Pseudomonas syringae* and had reduced SA accumulation (Song *et al.*, 2004a), while the overexpression of *ALD1* conferred resistance to the pathogen by inducing the expression of *PAD4* and *ICS1*, key components of the SA pathway that are both essential for the response (Cecchini *et al.*, 2015; Song *et al.*, 2004b). *FMO1*, another SAR regulatory gene, is also indispensable for systemic SA accumulation and SAR (Bartsch *et al.*, 2006; Koch *et al.*, 2006; Mishina and Zeier, 2006). *ALD1* transcript induction depends on *FMO1* during the establishment of SAR (Song *et al.*, 2004b). The *ALD1‐LIKE* gene of *Oryza sativa* (*OsALD1*) was shown to be involved in the resistance response of rice to the rice blast fungus (*Magnaporthe oryzae*) (Jung *et al.*, 2016). Interestingly, natural oviposition by *Pieris brassicae* or treatment with egg extract also induced SAR and inhibited growth of *P. syringae* in an *ALD1‐* and *FMO1*‐dependent manner, implicating the *ALD1*‐mediated pathway in a response to insects (Hilfiker *et al.*, 2014).

*ALD1* encodes an aminotransferase with multiple substrates and products *in vitro*. l‐lysine is used as a substrate by ALD1 to produce enaminic 2,3‐dehydropipecolic acid (2,3‐DP), and then (2,3‐DP) is converted to pipecolic acid (Pip) by SARD4 (Ding *et al.*, 2016; Hartmann *et al.*, 2017; Navarova *et al.*, 2012). Infection by *P. syringae* pv. *maculicola* (*Psm*) ES4326 induced a significant accumulation of Pip in the wild‐type, but not in *ald1* mutants. Pretreatment with Pip led to increased pathogen resistance in wild‐type plants, and exogenous Pip complemented the resistance defect of *ald1* mutants (Navarova *et al.*, 2012). Recently, it was reported that FMO1 can convert Pip to *N*‐hydroxypipecolic acid (NHP), which then regulates the systemic acquired resistance to pathogen infection (Chen *et al.*, 2018; Hartmann *et al.*, 2018). Moreover, in the pathway it was found that Pip triggers activation of MPK3 and MPK6 that regulates the transcription factor WRKY33 to promote the expression of *ALD1*, indicating the positive regulatory loop in the *ALD1*‐mediated resistance pathway (Wang *et al.*, 2018). Bernsdorff *et al*. investigated the relationships between SA and Pip, and showed that SA and Pip act independently from one another, but also synergistically in the basal immunity of *A. thaliana* to *P. syringae* (Bernsdorff *et al.*, 2016). The results suggest that Pip acts as an indispensable switch for the activation of SAR, and that SA amplifies, and is required for, Pip‐triggered responses (Bernsdorff *et al.*, 2016; Navarova *et al.*, 2012).

Meanwhile, exogenous Pip strongly induced resistance in the SA‐deficient *ics1* mutant, and both Pip and NHP pretreatment significantly increased the resistance of *sid2* plants to *Psm* and *Hpa*, although in all cases resistance was markedly lower than in wild‐type plants, indicating that an SA‐independent defence pathway probably functions simultaneously (Hartmann *et al.*, 2018; Navarova *et al.*, 2012). However, no such SA‐independent defence pathway has yet been identified.

The defence role of *ALD1* against bacterial pathogens has been well documented, and its function against fungal and oomycete pathogens has also been reported (Bernsdorff *et al.*, 2016; Jung *et al.*, 2016; Navarova *et al.*, 2012). Recent data showed that tobacco mosaic virus (TMV) infection led to increased Pip accumulation, and that Pip‐treated tobacco had smaller TMV lesions (Adam *et al*. 2018). We now report that the expression of *Nicotiana benthamiana ALD1* (*NbALD1*) is induced following infection by turnip mosaic virus (TuMV) and that *NbALD1* participates in plant defence against TuMV by both SA‐dependent and SA‐independent pathways simultaneously. We also show that the ethylene pathway, regulated negatively by *NbALD1* and mediating the susceptibility of *N. benthamiana* to TuMV, functions in *NbALD1* or Pip‐mediated resistance in an SA‐independent manner.

## Results

### Expression of *NbALD1* is induced in TuMV‐infected *N. benthamiana*

**Figure 1:**
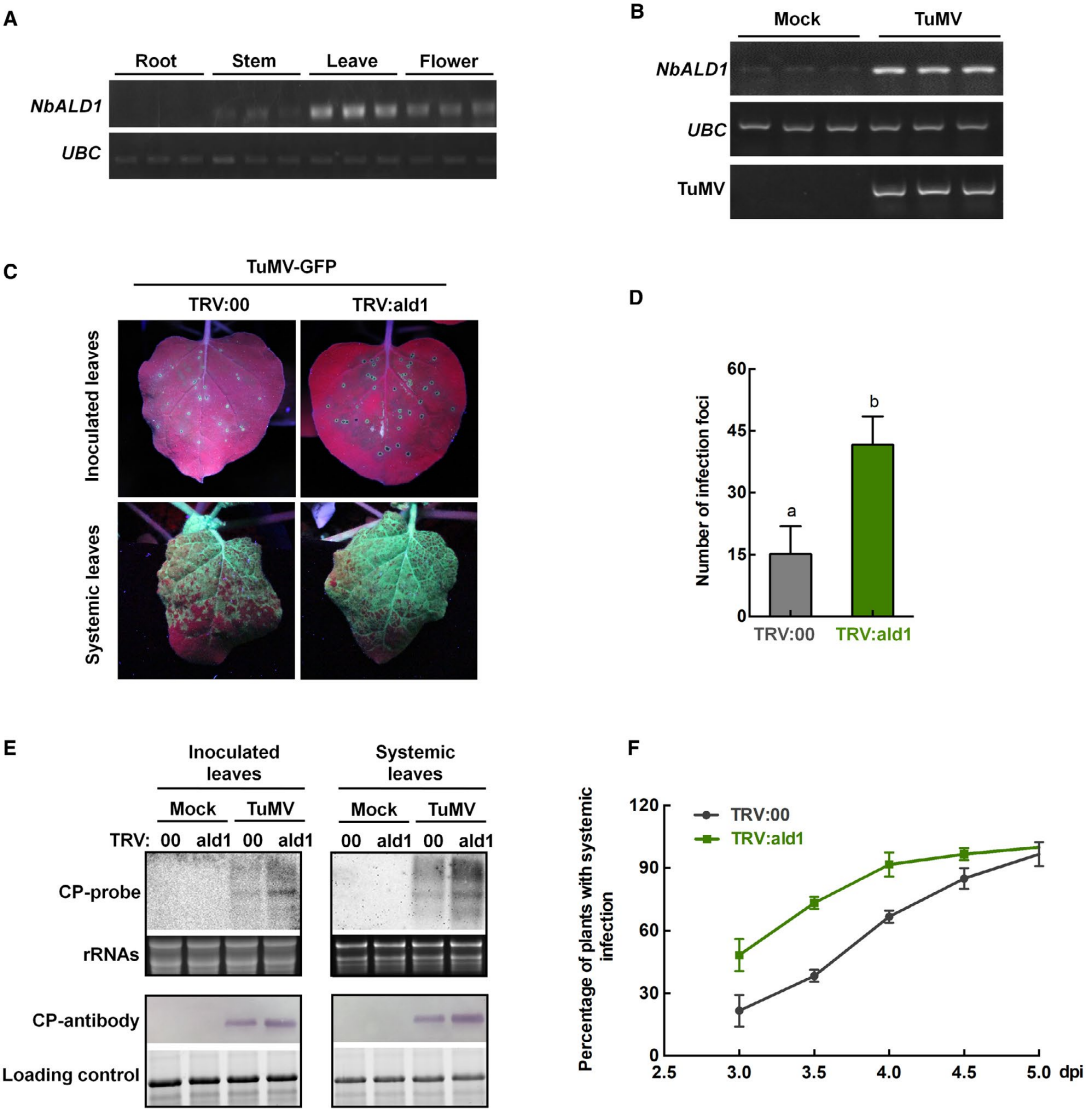
*NbALD1* was induced by TuMV in *N. benthamiana* and its silencing facilitated infection by TuMV. (A) Semi‐quantitative RT‐PCR showing that *NbALD1* was expressed at a higher level in leaves than in flowers, stems and roots. (B) Semi‐quantitative RT‐PCR showing that the expression of *NbALD1* in leaves was remarkably induced by TuMV infection at 5 dpi. Results from three independent biological replicates are shown; *Ubiquitin C* (*UBC*) was used as the internal reference gene. (C) Leaves of plants inoculated with TuMV‐GFP and examined under UV light at 4 dpi. On *NbALD1*‐silenced plants (TRV:ald1), there were more fluorescent spots (infection foci) on the inoculated leaves and a larger fluorescent area on systemically infected leaves than on the controls (TRV:00). (D) Numbers of infection foci on inoculated leaves of *NbALD1*‐silenced (TRV:ald1) and non‐silenced (TRV:00) plants at 4 dpi. Error bars show the mean ± SD of three replicates (at least 20 plants per replicate); different letters on histograms indicate significant differences (*P < *0.05). (E) Northern and western blots showing the increased accumulation of TuMV RNAs and CP protein in inoculated leaves and systemic leaves from *NbALD1*‐silenced plants (TRV:ald1) compared to the controls (TRV:00). (F) Percentage of plants with GFP fluorescence on newly emerged leaves (i.e. systemically infected) at different times after inoculation. Error bars show the mean ± SD of three replicates (at least 20 plants per replicate).

*Arabidopsis thaliana ALD1* (*AtALD1*, Accession No. NM_126957.2) was used as query sequence in a basic local alignment search tool (BLAST) of the *Nicotiana benthamiana* Genome v1.0.1 predicted cDNA database (https://solgenomics.net/tools/blast/) to identify its homolog in *N. benthamiana* (Sequence ID: Niben101Scf04547g02001.1; named *NbALD1*). *NbALD1* has 1350 nucleotides in an open reading frame encoding 450 amino acids. Sequence alignment showed that *NbALD1* protein had 60.9% identity to AtALD1 (Accession No. NP_565359.1) and 54.2–68.7% identities to the ALD1 or AGD2 of other plants (Fig. S1A,B). All ALD1s contained the conserved residues for the pyridoxal‐5ʹ‐phosphate (PLP)‐binding site and the malate binding site (Fig. S1B). *NbALD1* was expressed at a higher level in leaves than in flowers, stems or roots (Fig. 1A). At 5 days post inoculation (dpi) with TuMV, the expression of *NbALD1* in leaves of *N. benthamiana* was remarkably induced (Fig. 1B).

### Silencing of *NbALD1* facilitates infection by TuMV

To investigate the potential roles of *NbALD1* in TuMV infection, we used tobacco rattle virus (TRV)‐induced gene silencing (VIGS) to silence *NbALD1* and then inoculated TuMV onto the plants (Liu *et al.*, 2002). For this purpose, a sequence of 300 nt from *NbALD1* was inserted into TRV‐RNA2 to produce TRV:ald1, at 8 dpi the expression of *NbALD1* was only 18.4% of normal levels (Fig. S2). There were no obvious differences of phenotype between control (TRV:00) and *NbALD1*‐silenced plants (Fig. S2). Plants treated with TRV:ald1 or TRV:00 were then mechanically inoculated with green fluorescent protein (GFP)‐labelled TuMV (TuMV‐GFP) and subsequent viral infection was monitored by detecting GFP fluorescence. At 4 dpi, there were more fluorescent spots or infection foci on the inoculated leaves of *NbALD1*‐silenced plants (Fig. 1C,D). Both viral RNAs and coat protein (CP) accumulated much more in the inoculated leaves of *NbALD1*‐silenced plants than in the TRV:00‐treated controls (Fig. 1E). From 3 dpi, GFP fluorescence began to appear on newly emerged leaves, indicating systemic infection. Initially, the *NbALD1*‐silenced plants had a higher incidence of systemic infection and a larger area of GFP fluorescence than the controls, indicating a faster spread of systemic infection (Fig. 1C,F). Blotting analysis showed that viral RNAs and CP accumulated much more on the newly emerged leaves of silenced plants compared to the controls (Fig. 1E). Thus, silencing of *NbALD1* facilitated infection by TuMV.

### Overexpression of *NbALD1* confers enhanced resistance to TuMV

**Figure 2:**
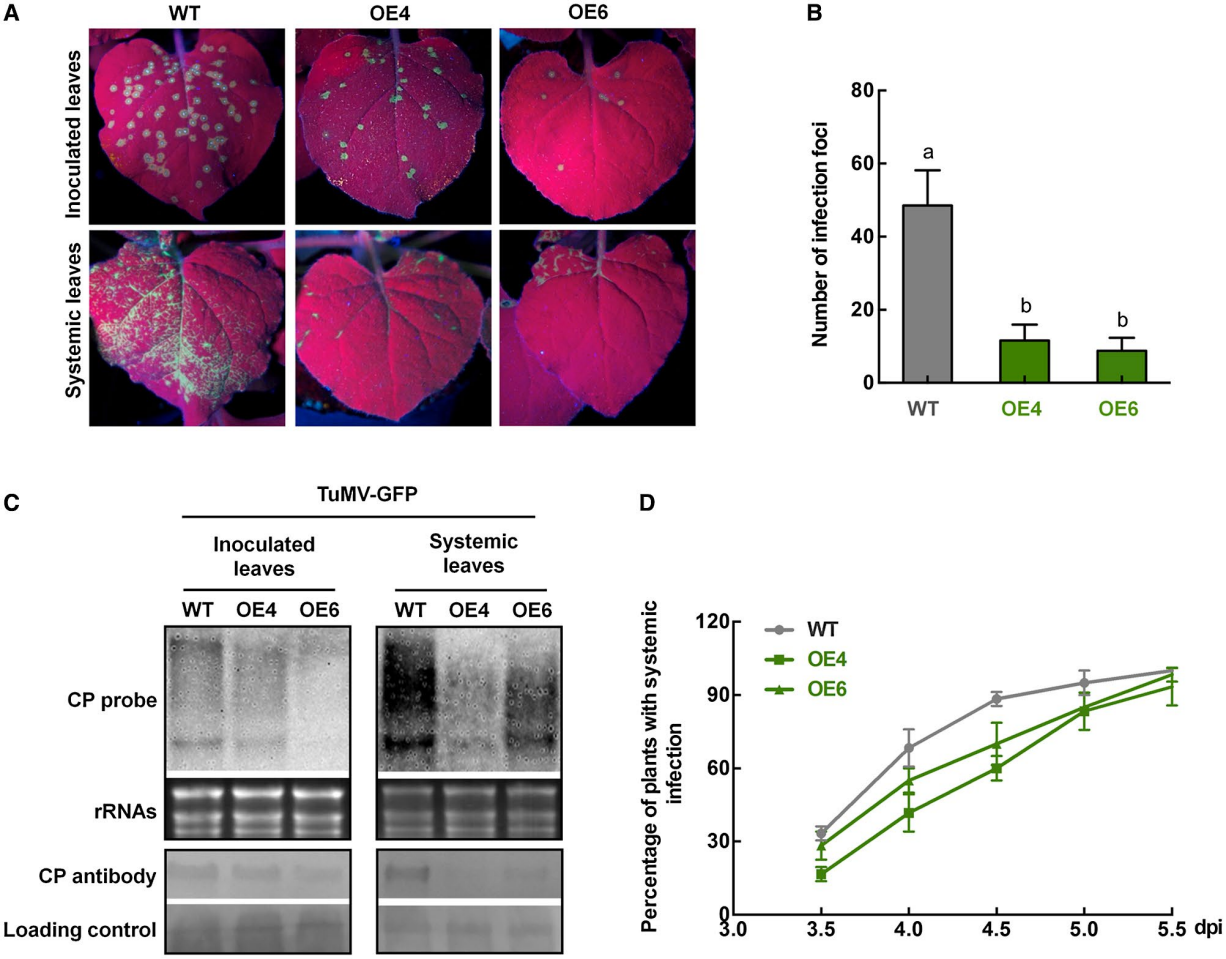
Overexpression of *NbALD1* enhanced *N. benthamiana* resistance to TuMV. (A) Leaves of plants inoculated with TuMV‐GFP and examined under UV light at 4 dpi. On plants overexpressing *NbALD1* (lines OE4 and OE6), there were fewer fluorescent spots (infection foci) on the inoculated leaves and a smaller fluorescent area on systemically infected leaves than on the wild‐type (WT) controls. (B) Numbers of infection foci on inoculated leaves from WT, OE4 and OE6 plants at 4 dpi. Error bars show the mean ± SD of three replicates (at least 20 plants per replicate); different letters on histograms indicate significant differences (*P < *0.05). (C) Northern and western blots showing that TuMV RNAs and CP protein accumulated less in both the inoculated and the newly emerged leaves of transgenic plants compared to the control WT plants. (D) Percentage of wild‐type and transgenic plants with GFP fluorescence on newly emerged leaves (i.e. systemically infected) at different times after inoculation. Error bars show the mean ± SD of three replicates (at least 20 plants per replicate).

To further determine the role(s) played by *NbALD1* in defence against TuMV, we obtained transgenic *N. benthamiana* expressing HA‐tagged *NbALD1* driven by the cauliflower mosaic virus (CaMV) 35S promotor (Fig. S3A). Transgenic plants had a similar phenotype to wild‐type at the early stage of development, but were slightly shorter at the flowering stage (Fig. S3B). Two lines of transgenic plants (OE4 and OE6) with increased levels of HA‐tagged *NbALD1* (Fig. S3C) were then inoculated with TuMV‐GFP. At 4 dpi, the number of infection foci was fewer on the inoculated leaves of transgenic plants than on the controls, and the fluorescence associated with systemic infection was also less extensive and less intense (Fig. 2A,B). Viral RNAs and CP accumulated less in both the inoculated and newly emerged leaves of transgenic plants than in control wild‐type plants (Fig. 2C) and the systemic infection spread more slowly (Fig. 2D). Thus, overexpression of *NbALD1* enhanced the resistance of *N. benthamiana* to TuMV.

### Exogenous application of pipecolic acid boosts *N. benthamiana* resistance to TuMV

**Figure 3:**
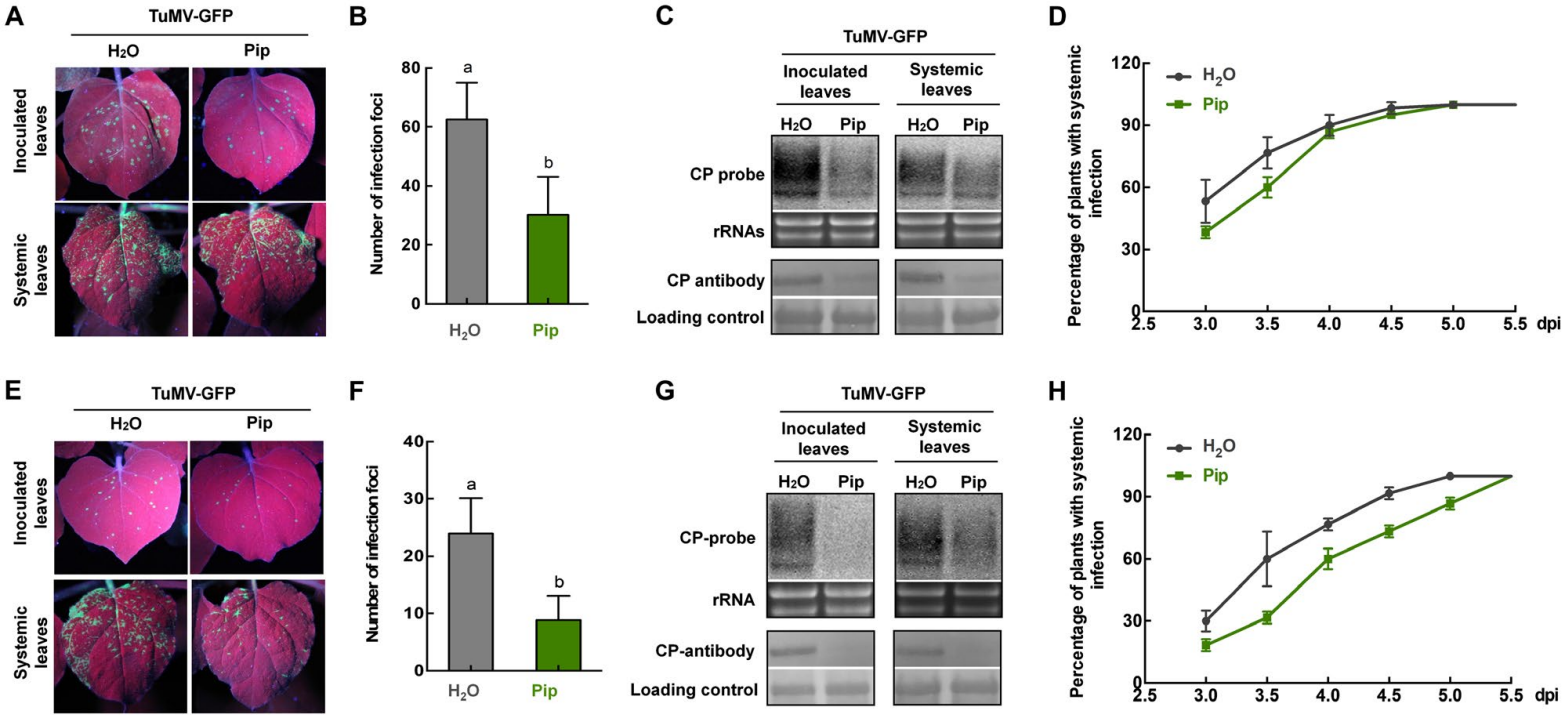
Exogenous application of pipecolic acid (Pip) enhanced *N. benthamiana* resistance to TuMV. (A) Leaves of plants inoculated with TuMV‐GFP on *NbALD1*‐silenced plants and examined under UV light at 4 dpi. (B) Numbers of infection foci on inoculated leaves from Pip‐ and H_2_O‐treated *NbALD1*‐silenced plants at 4 dpi. (C) Northern and western blots showing that TuMV RNAs and CP protein accumulated less in both the inoculated and the newly emerged leaves of Pip‐treated plants *NbALD1*‐silenced plants, compared to the H_2_O‐treated *NbALD1*‐silenced plants. (D) Percentage of plants systemically infected at different times after inoculation. (E) Leaves of plants inoculated with TuMV‐GFP on non‐silenced (TRV:00) plants and examined under UV light at 4 dpi. (F) Numbers of infection foci on inoculated leaves from Pip‐ and H_2_O‐treated non‐silenced (TRV:00) plants at 4 dpi. (G) Northern and western blots showing that TuMV RNAs and CP protein accumulated less in both the inoculated and the newly emerged leaves of Pip‐treated plants non‐silenced (TRV:00) plants, compared to the H_2_O‐treated non‐silenced (TRV:00) plants. (H) Percentage of plants systemically infected at different times after inoculation. Error bars show the mean ± SD of three replicates (at least 20 plants per replicate); different letters on histograms indicate significant differences (*P* < 0.05).

It has been reported that Pip, a downstream product of ALD1, is essential for *ALD1‐*mediated systemic acquired resistance and basal resistance (Navarova *et al.*, 2012). Exogenous application of Pip strongly boosted basal and specific resistance of both wild‐type and *ald1* mutant plants to compatible *Psm* and incompatible *Psm* avrRpm1 (Navarova *et al.*, 2012). Exogenous application of Pip also enhanced resistance of tobacco to the adapted *P. syringae* pv. *tabaci* (*Pstb*) or non‐adapted *Psm* (Vogel‐Adghough *et al.*, 2013). To confirm the defence role of *NbALD1* against TuMV, we treated *NbALD1*‐silenced *N. benthamiana* with 100 μM Pip for 8 days. Treated plants were then inoculated with TuMV‐GFP. At 4 dpi, there were fewer infection foci on Pip‐treated plants than on the water‐treated controls (Fig. 3A,B) and there were corresponding decreases in the accumulation of viral RNAs and CP in both inoculated and systemic leaves (Fig. 3C). Systemic infection also spread more slowly on Pip‐treated plants (Fig. 3D). Thus, Pip treatment alleviated the resistance deficiency of *NbALD1*‐silenced plants. Pip treatment also decreased the number of infection foci and the accumulation of TuMV on non‐silenced plants (Fig. 3E–H). Taken together, the results further confirm that *NbALD1* plays a role in defence against TuMV.

### SA accumulation is regulated positively by *NbALD1* and plays a role in defence against TuMV

**Figure 4:**
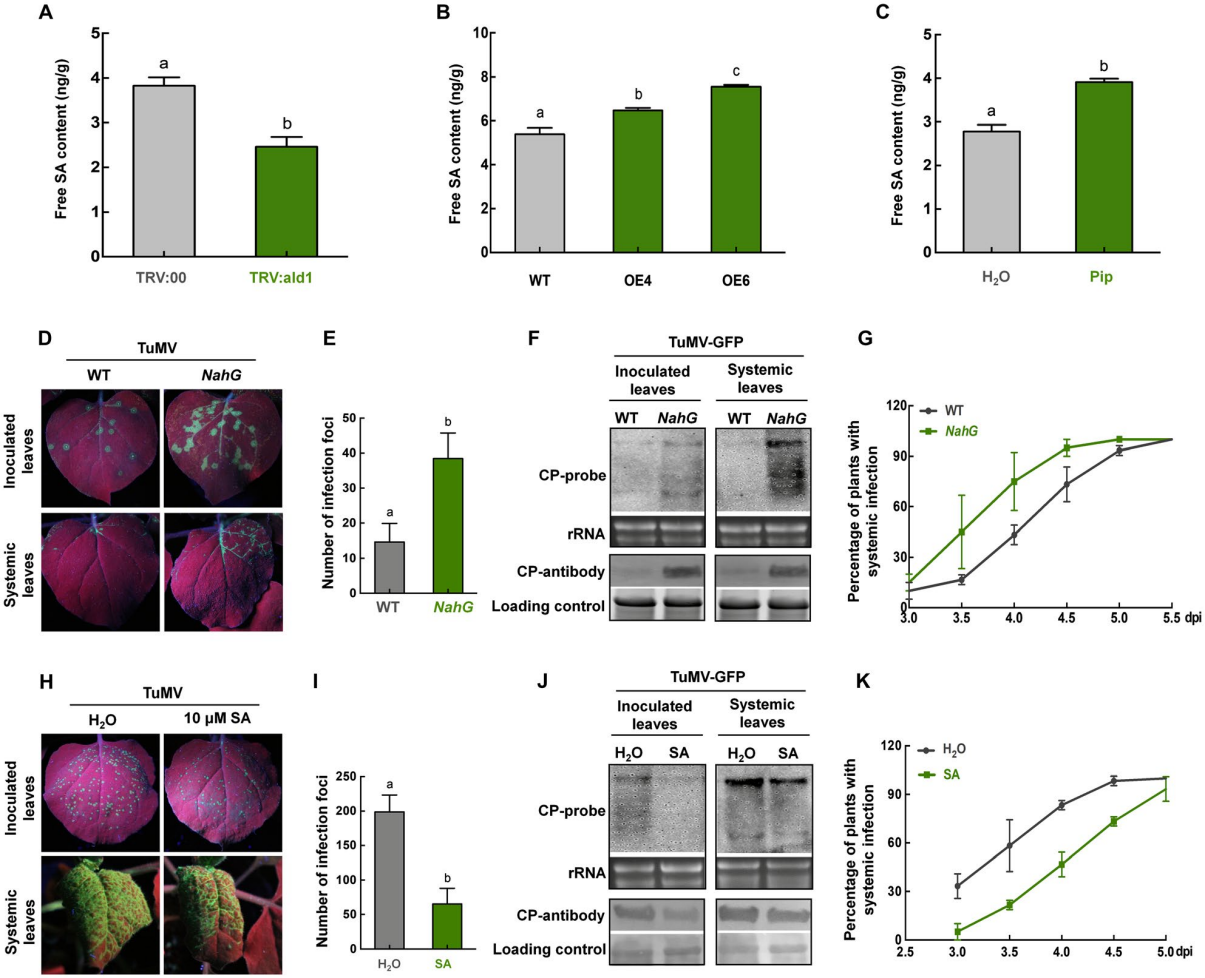
The SA accumulation was regulated positively by *NbALD1* and played a role in defense against TuMV. (A) SA content of *NbALD1*‐silenced (TRV:ald1) plants was significantly less than that in control plants (TRV:00). (B) SA content of *NbALD1* transgenic lines OE4 and OE6 was significantly greater than that in wild‐type (WT) plants. (C) SA content of 100 μM Pip‐treated WT plants was significantly greater than that in H_2_O‐treated control plants for 8 dpi. (D) Leaves of WT and transgenic NahG plants inoculated with TuMV‐GFP and examined under UV light at 4 dpi. (E) Numbers of infection foci on inoculated leaves. (F) Northern and western blots showing accumulation of TuMV RNAs and CP protein. (G) Percentage of plants systemically infected at different times after inoculation. (H) Leaves of H_2_O‐ and 10 μM SA‐treated WT plants inoculated with TuMV‐GFP and examined under UV light at 4 dpi. (I) Numbers of infection foci on inoculated leaves. (J) Northern and western blots showing accumulation of TuMV RNAs and CP protein. (K) Percentage of plants systemically infected at different times after inoculation. Error bars show the mean ± SD of three replicates (at least 20 plants per replicate). Different letters on histograms indicate significant differences (P < 0.05).

It has been reported that the *ALD1* or Pip‐induced plant defence responses to *P. syringae* are accompanied by increased SA (Bernsdorff *et al.*, 2016; Navarova *et al.*, 2012). We therefore measured the SA content of our plants where *NbALD1* was either silenced or overexpressed without virus infection. Compared to non‐silenced plants, the SA content of *NbALD1*‐silenced plants was decreased from 3.83 to 2.46 ng/g (Fig. 4A), but increased from 5.40 to 6.47 ng/g (OE4) or 7.55 ng/g (OE6) plants in those overexpressing *NbALD1* without TuMV infection (Fig. 4B). SA content in the Pip‐treated plants was also enhanced significantly (Fig. 4C). The results indicate that *NbALD1* positively regulates the SA content, similar to the previous reports on *ALD1*‐meditated resistance to bacterial pathogens.

We next examined whether the SA plays a role in defence to TuMV by inoculating TuMV‐GFP to *NahG* (which encodes a salicylate hydroxylase converting SA to catechol) transgenic *N. benthamiana* in which SA cannot accumulate. The *NahG* plants were more susceptible to virus than the controls by all measures: there were more infection foci, TuMV accumulated to greater levels on inoculated leaves (Fig. 4D–F), systemic infection spread more rapidly (Fig. 4G) and there was a greater accumulation of viral RNAs and CP (Fig. 4D–F). These results imply that the SA is involved in resistance to TuMV. As further confirmation, wild‐type plants were treated with 10 μM SA for 48 h and then inoculated with TuMV‐GFP. Compared to the controls, fewer infection foci appeared, viral RNAs and CP accumulated less and systemic infection occurred more slowly (Fig. 4H–K).

### The SA is an essential, but not the only, regulated pathway involved in *NbALD1*‐mediated resistance to TuMV

**Figure 5:**
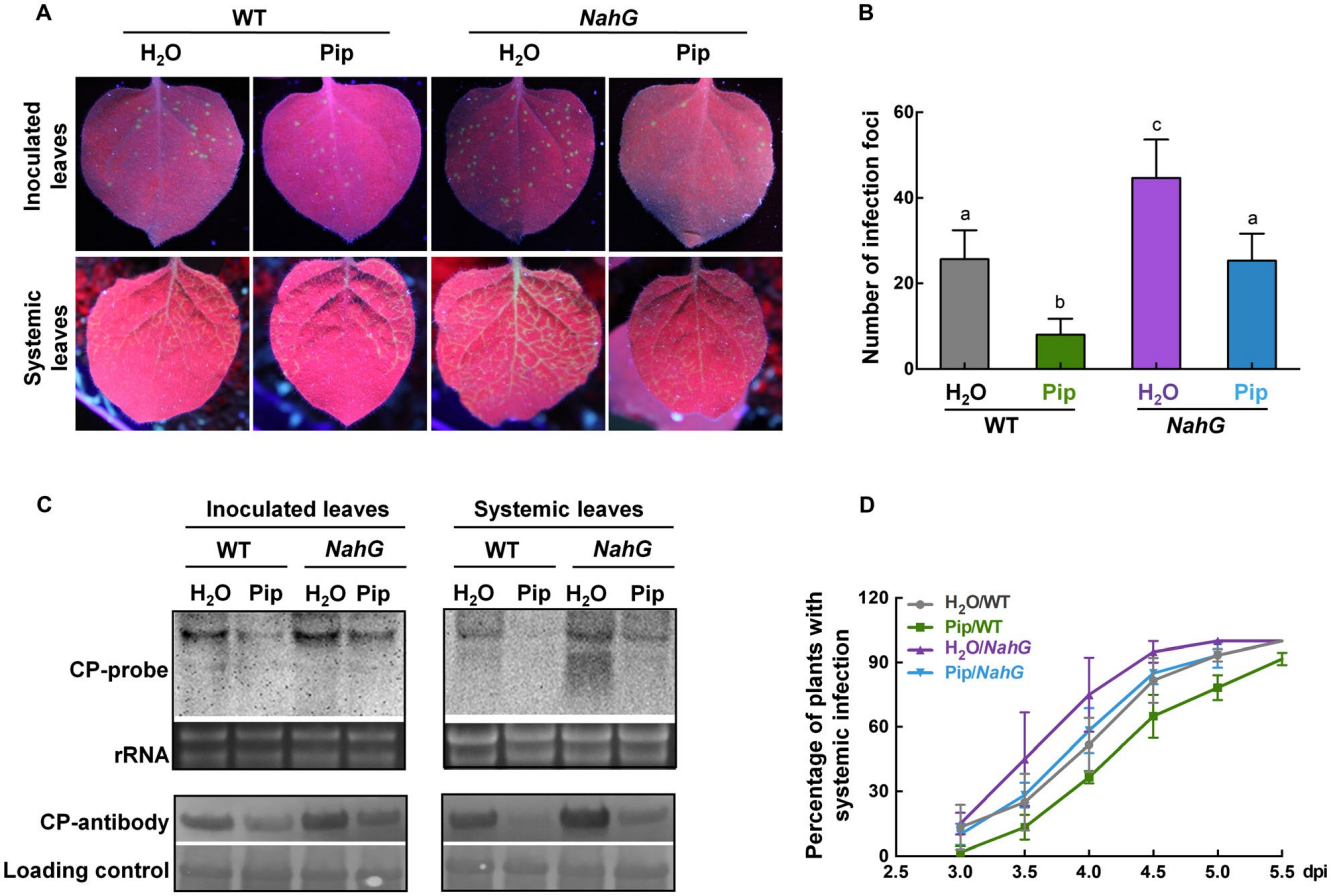
The SA was required but was not the only regulated pathway in *NbALD1*‐mediated resistance against TuMV. (A) Leaves of Pip‐treated wild type (WT) and *NahG* transgenic plants inoculated with TuMV‐GFP and examined under UV light at 4 dpi. (B) Numbers of infection foci on inoculated leaves at 4 dpi. Error bars show the mean ± SD of three replicates (at least 20 plants per replicate); different letters on histograms indicate significant differences (*P* < 0.05). (C) Northern and western blots showing the accumulation of TuMV RNAs and CP protein in the inoculated and systemic leaves. (D) Percentage of plants systemically infected at different times after inoculation. Error bars show the mean ± SD of three replicates (at least 20 plants per replicate).

To examine the association of the SA with *NbALD1*‐mediated resistance to TuMV, *NahG* plants were pretreated with Pip and then inoculated with TuMV‐GFP. The number of infection foci on inoculated leaves of Pip‐treated *NahG* plants was significantly less than on leaves of H_2_O‐pretreated *NahG* plants (Fig. 5A,B). The numbers were greater than on the corresponding wild‐type plants (Fig. 5A,B). TuMV RNAs and CP accumulated less in Pip‐treated *NahG* leaves than in control *NahG* leaves (Fig. 5C) and systemic infection on Pip‐pretreated *NahG* plants progressed more slowly than that on H_2_O‐treated *NahG* plants, but still faster than that on Pip‐treated wild‐type plants (Fig. 5D). Consequently, TuMV RNAs and CP accumulated less in systemic leaves of Pip‐pretreated *NahG* plants than in systemic leaves of H_2_O‐pretreated *NahG* plants, but more than in systemic leaves of Pip‐pretreated wild‐type plants (Fig. 5C).

We next investigated the role of SA on *NbALD1*‐silenced plants. Non‐silenced and *NbALD1*‐silenced plants were pretreated with water or SA and then inoculated with TuMV‐GFP. The number of infection foci on inoculated leaves of SA‐treated *NbALD1*‐silenced plants was significantly less than on leaves of water‐treated *NbALD1*‐silenced plants (Fig. S4A,B). TuMV RNAs and CP accumulated less in SA‐treated *NbALD1*‐silenced leaves than in water‐treated *NbALD1*‐silenced leaves (Fig. S4C), but not to the level found in water‐treated non‐silenced leaves. This indicates that exogenous SA partially complements the resistance deficiency of *NbALD1*‐silenced plants.

Thus, the absence of SA compromised the Pip‐ or *NbALD1*‐mediated resistance, indicating that SA was required for *NbALD1* to function effectively in resistance to TuMV. However, exogenous application of SA only partially attenuated the susceptibility caused by silencing of *NbALD1* and Pip treatment still alleviated the susceptibility of *NahG* plants to TuMV, suggesting that there might be a SA‐independent pathway also participating in the *NbALD1*‐mediated resistance against TuMV.

### The ethylene pathway is regulated negatively by *NbALD1* and mediates *N. benthamiana* susceptibility to TuMV

**Figure 6:**
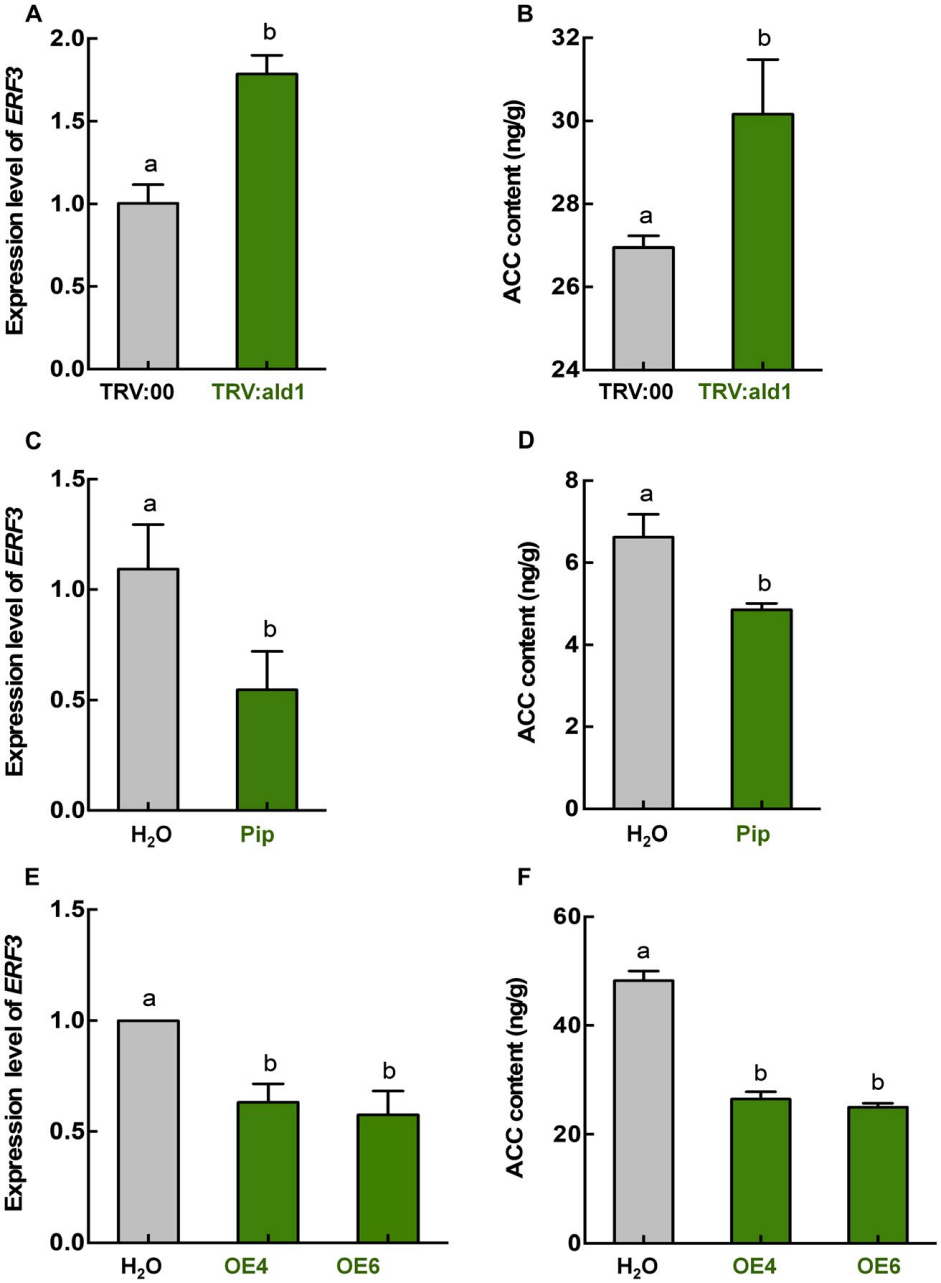
The Ethylene pathway was regulated negatively by *NbALD1*. (A) The increased expression of *NbERF3* in *NbALD1*‐silenced (TRV:ald1) plants. Empty TRV (TRV:00)‐treated plants were used as controls. (B) The increased ACC content in *NbALD1*‐silenced (TRV:ald1) plants. (C) The decreased expression of *NbERF3* in plants treated with 100 μM Pip. H_2_O‐treated plants were used as controls. (D) The decreased ACC content in plants treated with 100 μM Pip. (E) The decreased expression of *NbERF3* in *NbALD1* transgenic plants (OE4 and OE6). Wild type plants were used as controls. (F) The decreased ACC content in *NbALD1* transgenic plants (OE4 and OE6). Error bars show the mean ± SD of three replicates (at least 20 plants per replicate). Different letters on histograms indicate significant differences (*P* < 0.05).

Next we investigated whether a pathway other than SA might be involved in *NbALD1*‐mediated resistance to TuMV. An association between *ALD1* and the ethylene pathway has been reported in *Arabidopsis*: mutations in *ALD1* suppressed *edr2*‐mediated plant resistance to powdery mildew, programmed cell death and ethylene‐induced senescence, while an *ald1* single mutant had delayed ethylene‐induced senescence (Nie *et al.*, 2011). We therefore examined whether the ethylene pathway was involved in *NbALD1*‐mediated resistance to TuMV. The expression of ethylene‐responsive transcription factor 3 (*ERF3*), a key gene in the ethylene pathway, was significantly increased in *NbALD1*‐silenced plants (Fig. 6A) and there was increased accumulation of the precursor of ethylene, 1‐aminocyclopropanecarboxylic acid (ACC) (Fig. 6B). These results indicate the up‐regulation of the ethylene pathway in *NbALD1*‐silenced plants. Exogenous application of 100 μM Pip reduced the expression of *ERF3* and ACC content (Fig. 6C,D). In OE4 and OE6 plants overexpressing *NbALD1*, *ERF3* was remarkably less than in wild‐type plants, and ACC content was also less (48.3 ng/g in wild‐type, 26.5 ng/g in OE4 and 26.0 ng/g in OE6) (Fig. 6E,F). These results indicate that *NbALD1* is a negative regulator of the ethylene pathway.

**Figure 7:**
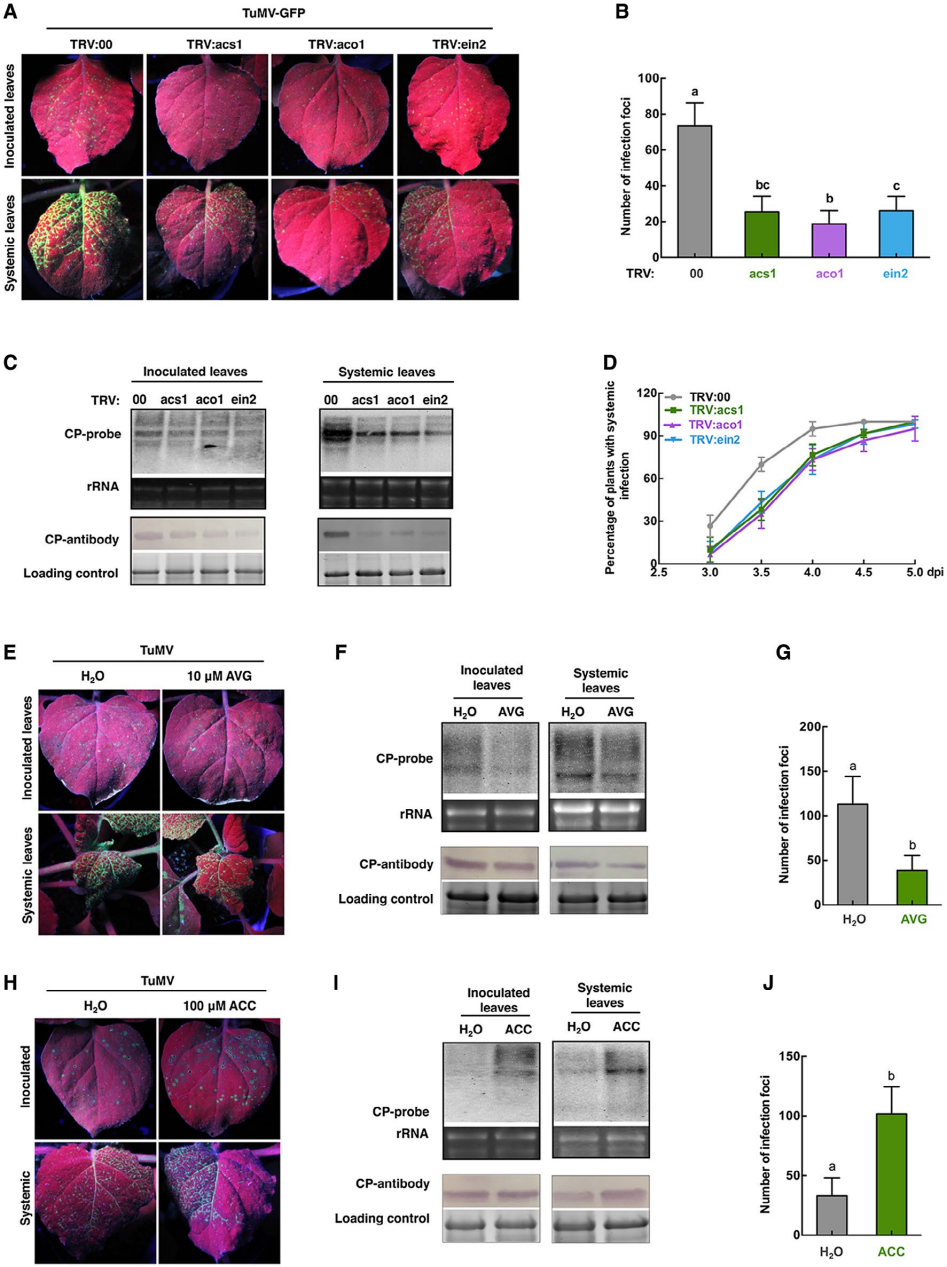
The ethylene pathway mediated susceptibility of *N. benthamiana* to TuMV. Silencing of three key genes in the ethylene pathway (*ACS1, ACO1* and *EIN2*) (A–D) or AVG treatment (E–G) increased resistance to TuMV while treatment with ACC increased susceptibility (H–J). (A, E and H) Leaves of plants inoculated with TuMV‐GFP and examined under UV light at 4 dpi. (B, G, and J) Numbers of infection foci on inoculated leaves at 4 dpi. Error bars show the mean ± SD of three replicates (at least 20 plants per replicate); different letters on histograms indicate significant differences (*P < *0.05). (C, F, and I) Northern and western blots showing accumulation of TuMV RNAs and CP protein. (D) Percentage of plants systemically infected at different times after inoculation. Error bars show the mean ± SD of three replicates (at least 20 plants per replicate).

The role of the ethylene pathway in TuMV infection was next examined. Key genes in the ethylene pathway, aminocyclopropane‐1‐carboxylic acid synthases 1(*ACS1*), ACC oxidase 1 (*ACO1*) and ethylene insensitive 2 (*EIN2*), were silenced by TRV‐mediated VIGS. At 14 dpi, the expression level of each gene was about 10% of normal levels (Fig. S5). TuMV‐GFP was then inoculated onto silenced plants. Compared to control leaves, there were fewer infection foci on inoculated leaves of silenced plants at 4 dpi of TuMV, and there was less accumulation of TuMV RNAs and CP (Fig. 7A–C). Systemic infection was also delayed, with corresponding decreases in the accumulation of TuMV RNAs and CP (Fig. 7C,D). These results indicate that plants were more resistant to TuMV when ethylene was suppressed. As further confirmation, we used aminoethoxyvinylglycine (AVG), an inhibitor of ethylene synthesis, to inhibit the ethylene pathway and examined its influence on TuMV infection. After spraying 10 μM AVG onto the leaves of *N. benthamiana*, expression of *ERF3* 16 h later was 72% of that in control plants sprayed with H_2_O (Fig. S6). After inoculation of the treated leaves with TuMV‐GFP there were fewer infection foci at 4 dpi than on the controls (Fig. 7E,G), and less accumulation of TuMV RNAs and CP (Fig. 7F). Thus, suppression of the ethylene pathway enhanced *N. benthamiana* resistance to TuMV.

To determine the influence of an up‐regulated ethylene pathway on TuMV infection, we sprayed 100 μM ACC on leaves and confirmed that *ERF3* was significantly up‐regulated 16 h later (Fig. S7). After inoculation of the ACC‐treated leaves with TuMV‐GFP, there were more infection foci at 4 dpi and much greater accumulation of viral RNAs and CP than on H_2_O‐treated control leaves (Fig. 7H–J), showing that up‐regulation of the ethylene pathway made *N. benthamiana* more susceptible to TuMV.

Taken together, the results suggest that the ethylene pathway was regulated negatively by *NbALD1* and mediated *N. benthamiana* susceptibility to TuMV.

### The negatively regulated ethylene pathway is involved in *NbALD1*‐mediated resistance against TuMV

**Figure 8:**
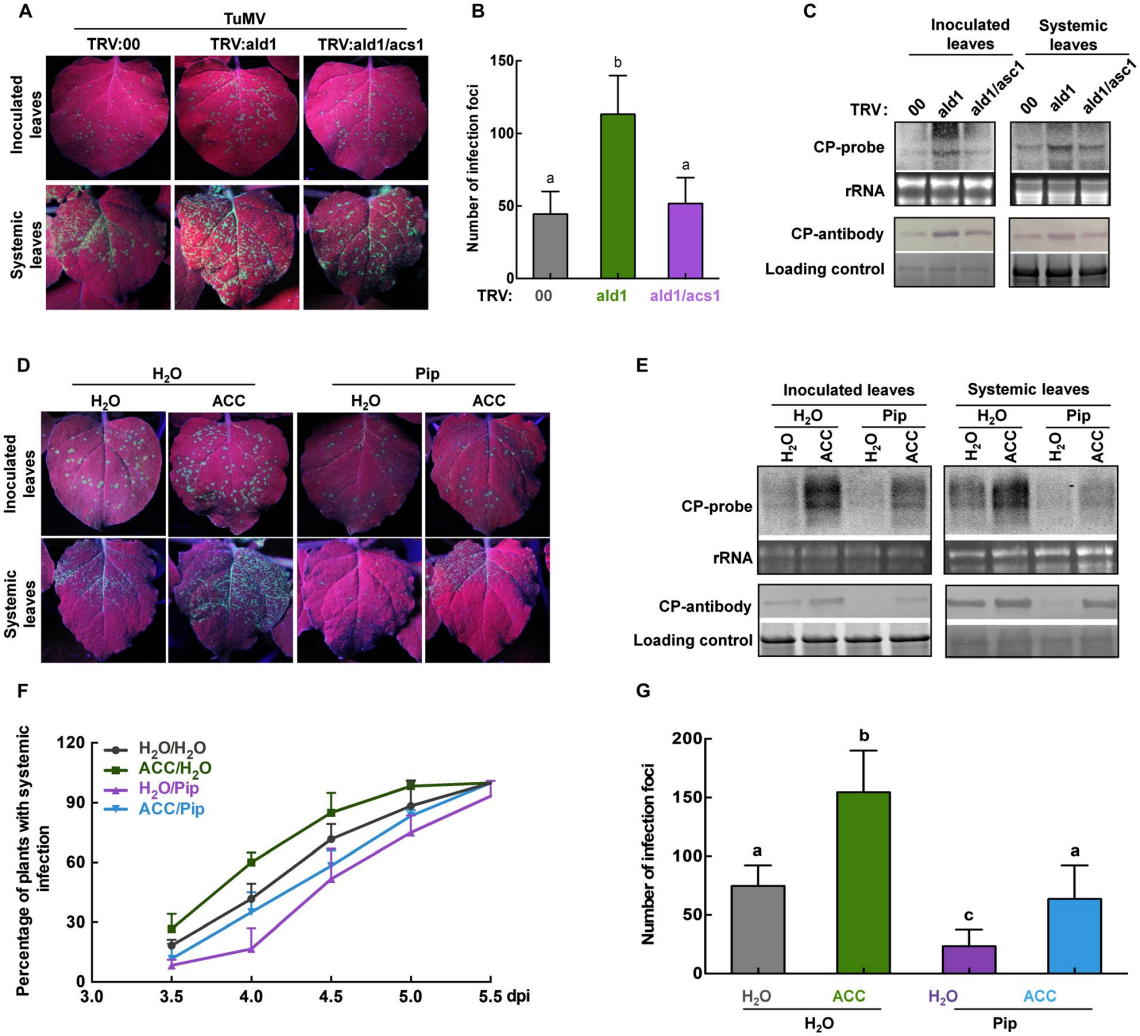
The negatively regulated ethylene pathway was involved in *NbALD1*‐mediated resistance to TuMV. (A) Leaves of plants inoculated with TuMV‐GFP and examined under UV light at 4 dpi. (B). Numbers of infection foci on inoculated leaves. (C) Northern and western blots showing accumulation of TuMV RNAs and CP protein. (D) Leaves of plants inoculated with TuMV‐GFP and examined under UV light at 4 dpi. (E) Northern and western blots showing accumulation of TuMV RNAs and CP protein. (F) Percentage of plants systemically infected at different times after inoculation. Error bars show the mean ± SD of three replicates (at least 20 plants per replicate); different letters on histograms indicate significant differences (*P* < 0.05). Silencing of *ACS1* enhanced the resistance of *NbALD1*‐silenced plants (A‐C) and ACC treatment compromised the resistance of Pip‐treated plants (D‐G).

The ethylene pathway was up‐regulated in plants where *NbALD1* was silenced and down‐regulated where it was overexpressed or if plants were treated with Pip (Fig. 6). To clarify the relationship between the ethylene pathway and *NbALD1*‐mediated resistance, we silenced *NbALD1* and *ACS1* simultaneously by VIGS for 8 days (Fig. S8) and then inoculated TuMV‐GFP onto silenced plants. The number of infection foci on inoculated leaves of *NbALD1/ACS1*‐silenced plants was about half that on *NbALD1*‐silenced leaves at 4 dpi (Fig. 8A and B) and there was much less accumulation of TuMV RNAs and CP (Fig. 8C). Because silencing of *NbACS1* lessened the susceptibility of *NbALD1*‐silenced plants to TuMV, it appears that the up‐regulated ethylene pathway may contribute to the susceptibility of *NbALD1*‐silenced plants to TuMV infection.

Finally, Pip‐treated wild‐type plants in which the ethylene pathway was down‐regulated were sprayed with 20 μM ACC and 16 h later were inoculated with TuMV‐GFP. The ACC spray treatment increased the number of infection foci at 4 dpi and there was a greater accumulation of TuMV RNAs and CP, indicating that exogenous application of ACC counteracted the resistance of Pip‐treated plants to TuMV (Fig. 8D–G).

## Discussion

Published evidence from *Arabidopsis* and rice suggests that the conserved plant ALD1 proteins may play a role in resistance to pathogens. *AtALD1* was induced by *P. syringae* infection in *Arabidopsis*, an *ald1*‐deficient mutant was hyper‐susceptible to the bacterium (Song *et al.*, 2004a) and overexpression of ALD1 inhibited the pathogen (Jong Tae *et al.*, 2004; Jung *et al.*, 2016)*.* The expression level of *OsALD1* increased when rice was infected by the fungus *M. oryzae* and overexpression of *OsALD1* conferred a remarkable resistance to the fungus (Jung *et al.*, 2016). Our finding that *NbALD1* in *N. benthamiana* was induced by TuMV infection and mediated the resistance to the virus supports the view that *ALD1* proteins have a conserved broad‐spectrum defence role in plants and provides the evidence of their activity against a viral pathogen.

The SA pathway plays a key role in *ALD1*‐mediated resistance to *P. syringae* in *Arabidopsis*. *Arabidopsis* overexpressing *ALD1* had increased SA content, and *ald1* mutants had decreased SA accumulation and were more susceptible to pathogens (Cecchini *et al.*, 2015; Song *et al.*, 2004a). Similar results were obtained from rice. Overexpression of *OsALD1* significantly induced the expression of pathogenesis‐related protein1 (PR1), the resistance gene responding to SA. It has recently been shown that SA modulates the accumulation of NHP (a downstream product), without affecting NHP generation. Our results found that silencing of *NbALD1* resulted in the reduction of SA content, while overexpression of *NbALD1* led to induction of SA in *N. benthamiana* (Fig. 4). SA has been reported to function in plant defence against viral pathogens. Silencing of SA biosynthetic and signalling genes in *N. benthamiana* plants increased susceptibility to TMV (Chivasa *et al.*, 1997). Exogenous application of SA or high levels of endogenous SA enhanced resistance to turnip crinkle viru*s* (TCV) (Chandra‐Shekara *et al.*, 2004). Citrus exocortis viroid (CEVd) or tomato spotted wilt tospovirus (TSWV) infection caused early and dramatic disease phenotypes in *NahG* tomato plants (Lopez‐Gresa *et al.*, 2016). Interestingly, HCPro, the RNA silencing suppressor of TuMV, interacts with AtCA1 (the homologue of SABP3 (SA‐binding protein 3)) to compromise the SA pathway and facilitate viral infection (Poque *et al.*, 2017). Recently, it was reported that high levels of guanosine pentaphosphate or tetraphosphate ((p)ppGpp) accumulation increased susceptibility to TuMV and reduced SA content, whereas plants with lower (p)ppGpp levels were more resistant to TuMV and had increased SA content, indicating a role for the SA pathway in defence against TuMV (Abdelkefi *et al.*, 2018). Our finding that transgenic *NahG N. benthamiana* was susceptible to TuMV, while exogenous SA enhanced *N. benthamiana* resistance to TuMV, demonstrates that SA participates in defence against TuMV in *N. benthamiana* (Fig. 4). SA accumulation is regulated positively by *NbALD1* and is essential for *NbALD1*‐mediated resistance to TuMV.

In examining the relationship between *NbALD1* and SA, we found that Pip treatment compromised the susceptibility of *NahG* plants to TuMV and also that SA treatment did not restore the resistance of *NbALD1*‐silenced plants to that of wild‐type plants, indicating that there are one or more unknown pathways regulated by *NbALD1* that participate in *NbALD1*‐mediated resistance to TuMV (Fig. 5). This is consistent with the results reported for the role of *ALD1* in *Arabidopsis* against *P. syringae*. In those experiments, treatment with Pip and NHP increased the resistance of wild‐type plants but also of the *sid2* mutant, suggesting that there is an SA‐independent role for *ALD1* in induced resistance (Bernsdorff *et al.*, 2016; Hartmann *et al.*, 2018; Navarova *et al.*, 2012; Vogel‐Adghough *et al.*, 2013), although the identity of that SA‐independent pathway was not determined.

Our experiments suggest that ethylene may be an important factor in *NbALD1*‐mediated resistance, at least to TuMV. Ethylene is an important gaseous phytohormone that affects plant growth, development and biotic stress (Cao *et al.*, 2007; Van Loon *et al.*, 2006; Wang *et al.*, 2013; Yang *et al.*, 2017). Silencing of *NbALD1* increased the content of ACC, the precursor of ethylene, and the expression level of *ERF3*, the ethylene response gene (Fig. 6). At the same time, Pip treatment or overexpression of *NbALD1* inhibited ACC accumulation and the expression of *ERF3* (Fig. 6). These results, therefore, indicate negative regulation of the ethylene pathway by *NbALD1*. However, the relationship between *ALD1* and ethylene may be complex because it has been reported that the *ald1* single mutant displayed an ethylene‐insensitive phenotype and delayed ethylene‐induced senescence (Nie *et al.*, 2011). Here, preliminary results showed that *ERF3* that was induced by TuMV infection on wild‐type plants was down‐regulated in TuMV‐infected plants overexpressing *NbALD1*, while in *ACS*‐silenced plants expression of *NbALD1* was still up‐regulated by TuMV infection (Fig. S9). We suppose that there may be a complicated mechanism to manipulate the regulation of ALD1 and balance the levels of ethylene and salicylic acid, which is worth investigating further.

Increasing evidence shows that ethylene plays roles in viral infection. The symptoms of chlorosis and stunting shown by plants infected with CaMV or expressing CaMV‐P6 (the main symptom determinant protein) depended on interactions between P6 and ethylene‐associated components (Geri *et al.*, 2004), while *Arabidopsis* mutants with defects in ethylene signalling showed reduced CaMV susceptibility (Love *et al*., 2007). Silencing ethylene biosynthetic and signalling genes strongly increased susceptibility to Chili veinal mottle virus (ChiVMV), indicating a key role for ethylene in plant systemic resistance against ChiVMV (Zhu *et al.*, 2014). Ethylene‐inducible transcription factor RAV2 is required for suppression of RNA silencing by two unrelated plant viral proteins, potyvirus HC‐Pro and carmovirus P38 (Endres *et al.*, 2010). While efficient infection by rice dwarf virus (RDV) depends upon a specific interaction between RDV‐Pns11 protein and OsSAMS, a key component of ethylene biosynthesis (Marco *et al.*, 1976; Zhao *et al.*, 2017), TuMV infection induce ethylene to benefit the virus (Casteel *et al.*, 2015). These reports indicate a complicated role for ethylene during viral infection. In our experiments, silencing of genes in the ethylene pathway, *ACS1*, *ACO1* and *EIN2*, increased plant resistance against TuMV (Fig. 7). Consistent with this, treatment with AVG, an inhibitor of ethylene, also enhanced plant resistance to TuMV (Fig. 7), while treatment with ACC made plants more susceptible (Fig. 7). The results thus demonstrate that the ethylene pathway is associated with plant susceptibility to TuMV and may be necessary for TuMV infection. Additionally, silencing of *ACS1* in the ethylene pathway alleviated the susceptibility of *NbALD1*‐silenced plants to TuMV (Fig. 8), while exogenous application of ACC compromised the resistance of Pip‐treated plants and *NbALD1* transgenic plants to TuMV (Fig. 8). These results demonstrate that the ethylene pathway is negatively regulated by *NbALD1* and functions as an active defence against TuMV infection.

Taken together, the results reported here suggest a model for the role of *NbALD1* in defence against TuMV that involves two regulated pathways: SA mediates resistance to TuMV and is positively regulated by *NbALD1*, while the ethylene pathway mediates susceptibility to the virus and is negatively regulated by *NbALD1*. It would be worth examining the possible relationship between these two pathways in *NbALD1*‐mediated resistance against TuMV.

## Experimental procedures

### Plants and virus inoculation

*N. benthamiana* plants and *NahG* transplants were grown in a glasshouse as previously described (Shi *et al.*, 2016). Plant at 5 weeks old were chosen to be infiltrated with *Agrobacterium tumefaciens* carrying TuMV‐GFP vector. At 6 dpi, the systemically infected leaves were collected for 100 mg and ground in 10 mL PBS buffer (8 *g*/L NaCl, 0.2 *g*/L KCl, 1.44 *g*/L Na_2_HPO_4_, 0.24 *g*/L KH_2_PO_4_, pH 7.4), and suspended to use for mechanical virus inoculation. Then plants at 3 weeks old were used for mechanical virus inoculation. Plants were examined daily and the numbers with symptoms recorded. After the symptoms appeared on the upper leaves, the leaves were photographed and harvested. The number of infection foci was assessed on 20 plants for each treatment (Table S1).

### Vector construction

For overexpressing *NbALD1*, the entire open reading frame (ORF) of *NbALD1* was fused with HA‐tag and then introduced into the PCV vector. The inserted fragment was obtained by PCR using a pair of primers linked to *Bam*HI and *Sac*I sequences on either side. The PCV vector was digested with *Bam*HI and *Sac*I, then the digested vector and PCR product were treated with T4 DNA ligase. To suppress *NbALD1*, a segment of *NbALD1* was obtained by PCR and then ligated into the TRV‐RNA2 vector as previously described (Liu *et al.*, 2002). TRV‐RNA2 was digested with *Pst*I, then the digested PCR and PCR products were treated with T4 DNA polymerase in the presence of dTTP and dATP. The resulting constructs were verified by sequencing and transformed to *Agrobacterium tumefaciens* strain GV3101. All primers used are listed in Table S2.

### Exogenous application of Pip, SA, AVG and ACC

Ten millilitres of a 100 μM L‐Pip (P1404; TCI Europe) solution was pipetted onto the soil substrate of individually cultivated plants, 8 days prior to virus inoculation. SA was applied to leaves at a concentration of 10 μM and these leaves were inoculated with virus 48 h later. AVG and ACC were sprayed onto leaves at concentrations of 10 or 100 μM respectively, and leaves were inoculated with virus 16 h later. In all cases, plants were similarly treated with water as controls.

### Quantification of SA and ACC from tobacco leaves

SA content was determined from 0.5 *g* samples of fresh leaves ground in liquid nitrogen using a mortar and pestle. After the addition of 5 mL exaction buffer (isopropanol/hydrochloric acid), the suspension was stirred for 30 min at 4 °C, 10 mL dichloromethane was added and the mixture stirred again for 30 min at 4 °C. After centrifugation at 13 000 *g* for 5 min at 4 °C, the supernatants were pooled, the organic phase was dried with nitrogen and then dissolved in 400 μL methanol containing 0.1% carboxylic acid. Samples were analysed by HPLC‐electrospray ionization/MS‐MS using a Waters ACQUITY UPLC coupled to an Xevo TQ. Chromatographic separation was carried out on a Waters ACQUITY UPLC R BEH C18 100 mm × 2.1 mm × 1.7 μm column at 40 °C. The solvent gradient used was 100% A (98/2 = H_2_O/CH_3_OH (V/V) +0.05% CHOOH + 5 mM CH_3_COONH_4_) to B (CH_3_CN) over 10 min. Solvent B was held at 100% for 5 min then the solvent returned to 100% A for 10 min equilibration prior to the next injection. The solvent flow rate was 0.3 mL/min. The MS was operated in the negative mode using electro‐spray ionization as the ion source.

ACC was extracted from the same leaf tissues which were ground in liquid nitrogen, then transferred into 50 mL microfuge tubes with 5 mL deionized water and treated in the ultrasonic cleaning instrument for 20 min. After centrifugation at 10 000 *g* for 5 min at 4°C, the supernatants were collected (pH = 4.0). Twenty millilitres of trichloromethane was added, the mixture was centrifuged at 10 000 *g* for 5 min and the supernatants were collected and infiltrated through a MCX polybase which had been pre‐activated by 3 mL CH_3_OH and 3 mL deionized water. The residue was eluted with 4 mL 1 M NH_3_·H_2_O. The ACC concentration in the supernatant was determined by HPLC‐electrospray ionization/MS‐MS.

SA and ACC assays used three replicates each of at least ten plants per treatment and genotype.

### Northern blot, quantitative RT‐PCR and semi‐quantitative RT‐PCR

Total RNA was extracted from plants with TRIzol (Invitrogen, Carlsbad, CA, USA) according to the manufacturer’s instructions. For northern blot, 10 μg of total RNA was separated on 1.5% formaldehyde agarose gels in 1 × MOPS buffer, and blotted onto Hybond‐N + membranes (GE Healthcare, Chicago, IL, USA). A probe of nearly 300 bp partially complementary to TuMV was labelled with Digoxigenin (DIG) (Roche, Basel, Switzerland). The sequences of the primers used are listed in Table S2.

For reverse transcription polymerase chain reaction (RT‐PCR), 1 μg of total RNA was reverse transcribed into cDNA by PrimeScript RT Enzyme (Takara). *N. benthamiana Ubiquitin C* gene (*UBC*) gene (Accession No. AB026056.1) was used as the internal reference gene for analysis. A Vazyme Ace qRT‐PCR system was used for the reaction and the results were analysed by the **∆∆**C_T_ method. The primer sequences used for this assay are listed in Table S2.

The *UBC* gene was also used as the internal reference gene in semiquantitative RT‐PCR analysis. Reactions were subjected to a varying number of cycles (28, 30, 32, 34, 36 and 38) of PCR amplification to determine the optimal cycle number within the linear range for PCR amplification. Products collected at various cycles were analysed by electrophoresis in 1.5% agarose‐GelRed gels, and those at the optimal cycle for each gene were used to compare relative expression levels. Primer sequences used in this assay are listed in Table S2.

### Western blot

Total proteins were extracted from plant samples using lysis buffer (100 mM Tris‐HCl, pH 8.8, 60% SDS, 2% β‐mercaptoethanol) and separated in a 12% SDS‐PAGE gel as previously described (Jiang *et al.*, 2014), then transferred onto nitrocellulose (Amersham, Uppsala, Sweden) by electroblotting, and detected with primary antibody to GFP and HA‐tag and secondary anti‐mouse and anti‐rabbit antibodies (Sigma‐Aldrich, St Louis, MO, USA). The antigen‐antibody (for secondary anti‐mouse) complexes were visualized using NBT/BCIP buffer (Sigma) at room temperature and the antigen‐antibody (for secondary anti‐rabbit) was visualized using ECL HRP Chemiluminescent Substrate (Invitrogen) under a chemiluminescence analyzer.

### Quantification and statistical analysis

Three replicates, each of 20 plants per treatment and genotype, were used to assess the rate of systemic infection (Table S3) and three independent plants per treatment and genotype were sampled for qRT‐PCR analyses in all experiments. All results were confirmed by three independent experiments. ANOVA analyses with type II sum of squares were performed on log 10‐transformed values to assess statistical differences of datasets. Unpairwise comparisons of treatment versus control values were performed with a two‐tailed Student’s *t*‐test in Prism 6, and Holm–Sidak's multiple comparisons test was used for multiple comparison.

## Supporting information


**Fig. S1** Identities and alignment among ALD1 from different plants.

**Fig. S2** Silencing of *NbALD1* in *N. benthamiana*.

**Fig. S3** Overexpression of *NbALD1* in *N. benthamiana*.

**Fig. S4** Exogenous SA partially complemented the resistance deficiency of *NbALD1*‐silenced plants.

**Fig. S5** Silencing of *ACS1*, *ACO1* and *EIN2* in *N. benthamiana*.

**Fig. S6** AVG treatment inhibited the expression of *ERF3*.

**Fig. S7** ACC treatment induced the expression of *ERF3*.

**Fig. S8** Silencing of *NbALD1* and *ACS1* in *N. benthamiana* simultaneously.

**Fig. S9** Expression of *ERF3* in TuMV‐infected wild‐type plants (A), *NbALD1*‐overexpressed plants (B) and expression of *NbALD1* in TuMV‐infected *ACS1*‐silenced plants (C).

**Table S1** Numbers of infection loci and statistical analysis.

**Table S2** Primers used for analysis.

**Table S3** Numbers of plants showing fluorescence under UV light in the systemic leaves at different times and statistical analysis of infection rates.
