# Supplementary material for: NbALD1 mediates resistance to turnip mosaic virus by regulating the accumulation of salicylic acid and the ethylene pathway in Nicotiana benthamiana
Source: Mol Plant Pathol. 2019 Apr 23;20(7):990–1004. doi: 10.1111/mpp.12808 (PMC6589722; doi:10.1111/mpp.12808)
Supplement: Supplementary file 1 — Fig. S1 Identities and alignment among ALD1 from different plants. [file MPP-20-990-s001.docx]

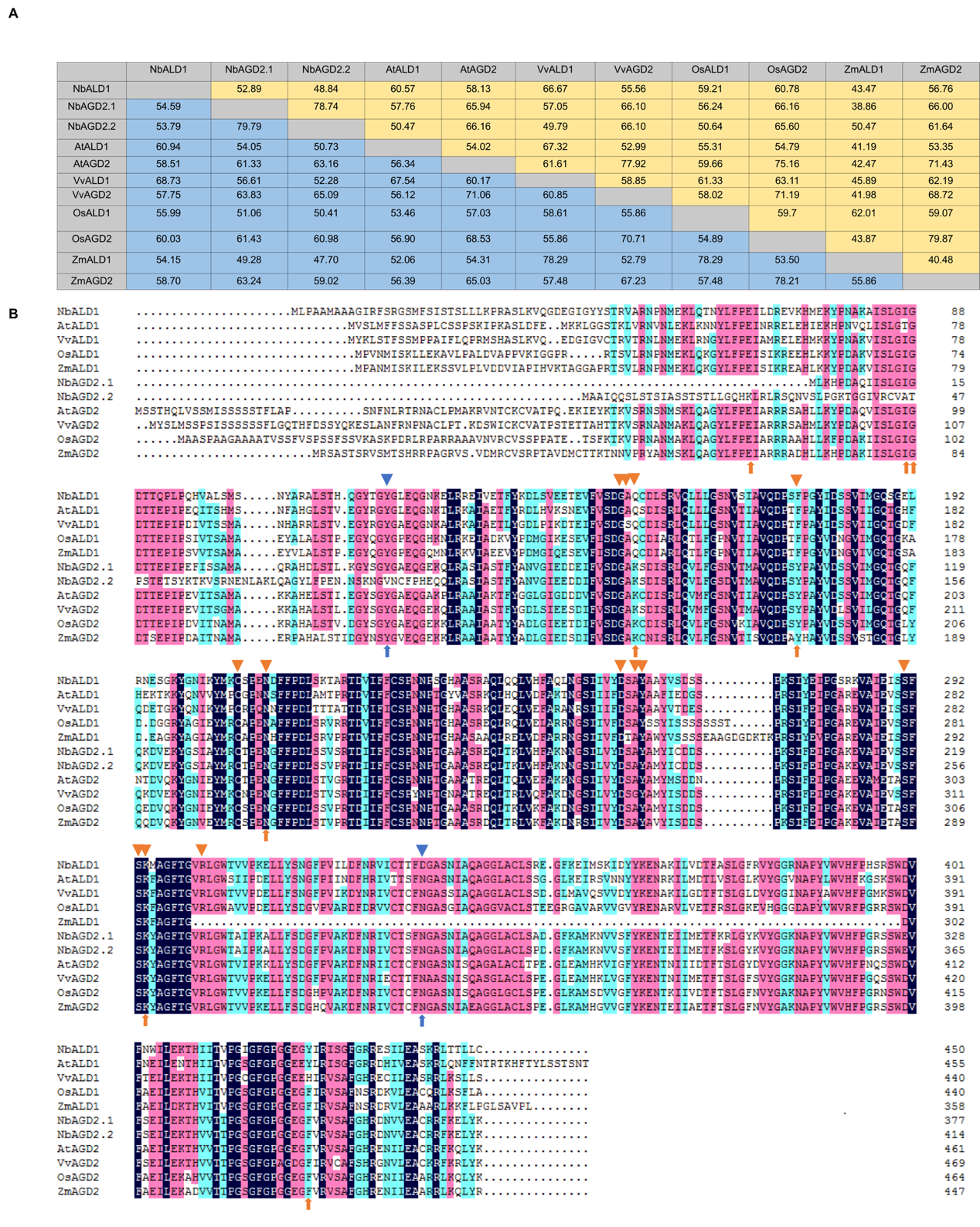


**Fig. S1 Identities and alignment among ALD1 from different plants.**

A. Amino acid (light brown) and nucleotide (light blue) identities between *NbALD1* and the *ALD1* and *AGD2* sequences of other plants.

B. Amino acid alignment of NbALD1 with the   ALD1s and AGD2s from other plants. Triangles show the PLP-binding sites and arrows represent the malate-binding sites (orange: subunit A; blue, subunit B). The *ALD1* and *AGD2* sequences used were as follows: *NbALD1* (Sequence ID: Niben101Scf04547g02001.1, https://solgenomics.net), *AtALD1* (NM_126957.2) and *AtAGD2* (NM_119526.4) from *Arabidopsis*, *OsALD1* (XM_015775297.2) and *OsAGD2* (XM_015775955.2) from *Oryza* *sativa*, *VvALD1* (XM_002268730.3), *VvAGD2* (FN595512.1) from *Vitis vinifera*, *ZmALD1* (XM_008654283.3) and *ZmAGD2* (GU180091.1) from *Zea mays* (dark blue: 100% of the homology level, pink: more than 75% of the homology level, light blue: more than 50% of the homology level).
