# Supplementary material for: NbALD1 mediates resistance to turnip mosaic virus by regulating the accumulation of salicylic acid and the ethylene pathway in Nicotiana benthamiana
Source: Mol Plant Pathol. 2019 Apr 23;20(7):990–1004. doi: 10.1111/mpp.12808 (PMC6589722; doi:10.1111/mpp.12808)
Supplement: Supplementary file 2 — Fig. S2 Silencing of NbALD1 in N. benthamiana. [file MPP-20-990-s002.docx]

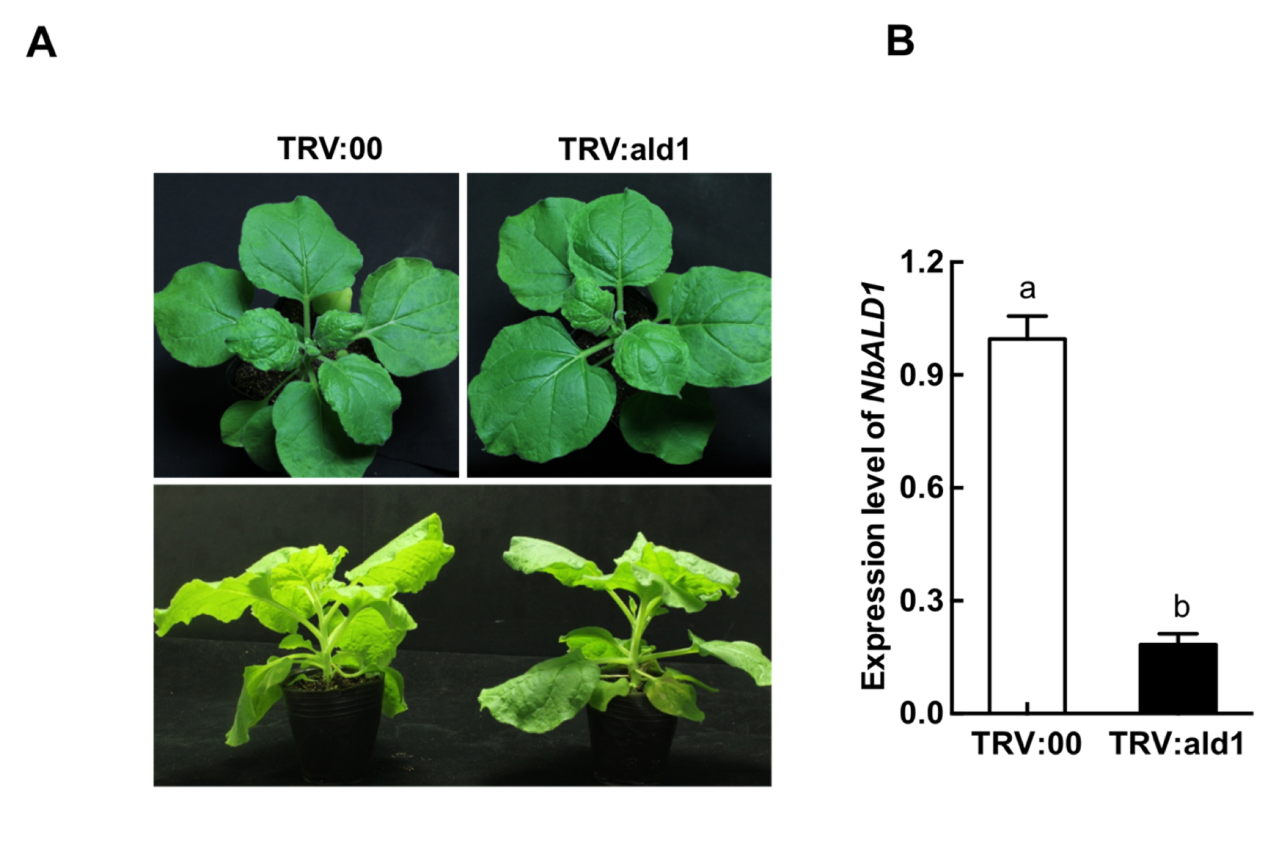


**Fig. S2 Silencing of *NbALD1* in *N. benthamiana***

A, The phenotype of non-silenced (TRV:00) and *NbALD1*-silenced (TRV:ald1) plants. Photos were taken under bright light 8 days after inoculation. B. Results of qRT-PCR showing the decreased expression of *NbALD1* in TRV:ald1-treated plants. Error bars show the mean ± SD of three replicates (at least 20 plants per replicate). Different letters on histograms indicate significant differences (*p <* 0.05).
