# Supplementary material for: NbALD1 mediates resistance to turnip mosaic virus by regulating the accumulation of salicylic acid and the ethylene pathway in Nicotiana benthamiana
Source: Mol Plant Pathol. 2019 Apr 23;20(7):990–1004. doi: 10.1111/mpp.12808 (PMC6589722; doi:10.1111/mpp.12808)
Supplement: Supplementary file 3 — Fig. S3 Overexpression of NbALD1 in N. benthamiana. [file MPP-20-990-s003.pdf]

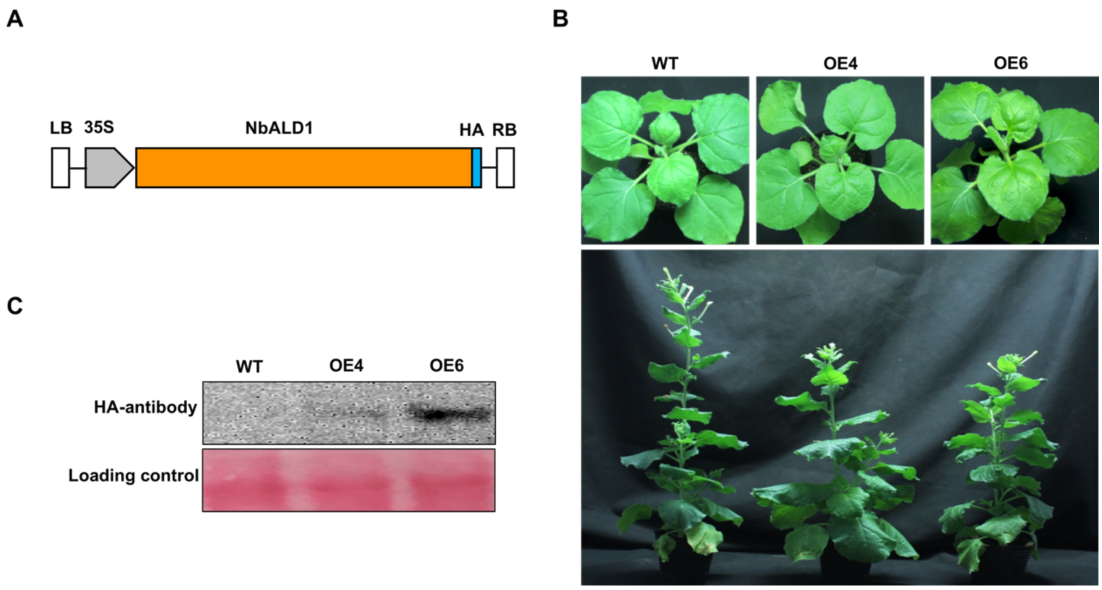

Fig. S3 Overexpression of *NbALD1* in *N. benthamiana*

A. Diagram of the pCV-35S plant expression vector containing HA-tag-fused *NbALD1*. LB is the left border and RB is the right border of the T-DNA. 35S is the 35S promoter encoded by CaMV. B. The phenotype of WT, OE4 and OE6 plants. Photos were taken under bright light at 8-weeks old. C. The expression of HA-tag-fused NbALD1, detected by HA-tag antibody. Ribisco proteins were used as loading controls.
