# Supplementary material for: NbALD1 mediates resistance to turnip mosaic virus by regulating the accumulation of salicylic acid and the ethylene pathway in Nicotiana benthamiana
Source: Mol Plant Pathol. 2019 Apr 23;20(7):990–1004. doi: 10.1111/mpp.12808 (PMC6589722; doi:10.1111/mpp.12808)
Supplement: Supplementary file 4 — Fig. S4 Exogenous SA partially complemented the resistance deficiency of NbALD1‐silenced plants. [file MPP-20-990-s004.docx]

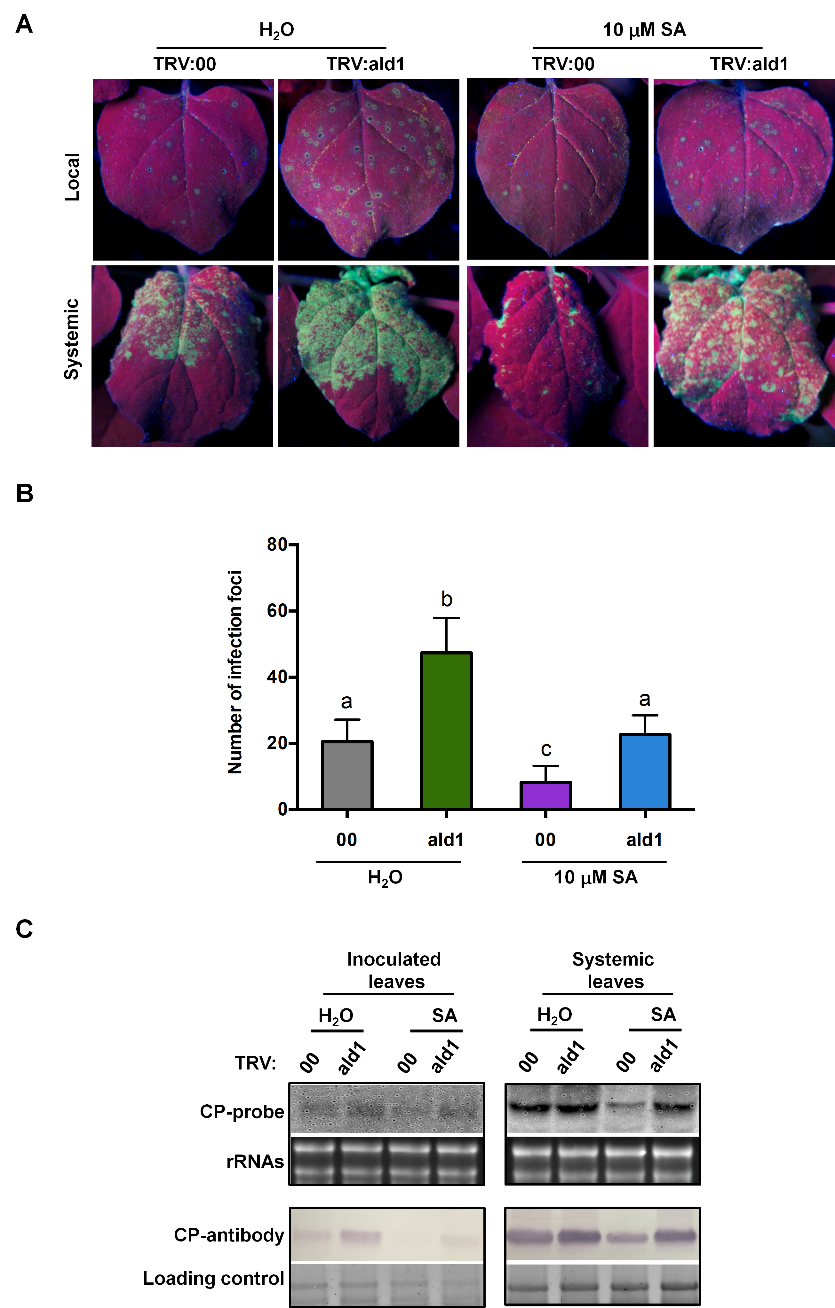


**Fig. S4** **Exogenous SA partially complemented the resistance deficiency of *NbALD1*-silenced plants.**

Leaves of plants inoculated with TuMV-GFP and examined under UV light at 4 dpi. B. Numbers of infection foci on inoculated leaves. Error bars show the mean ± SD of three replicates (at least 20 plants per replicate); different letters on histograms indicate significant differences (*p <* 0.05). C. Northern and western blots showing accumulation of TuMV RNAs and CP protein.
