# Supplementary material for: NbALD1 mediates resistance to turnip mosaic virus by regulating the accumulation of salicylic acid and the ethylene pathway in Nicotiana benthamiana
Source: Mol Plant Pathol. 2019 Apr 23;20(7):990–1004. doi: 10.1111/mpp.12808 (PMC6589722; doi:10.1111/mpp.12808)
Supplement: Supplementary file 5 — Fig. S5 Silencing of ACS1, ACO1 and EIN2 in N. benthamiana. [file MPP-20-990-s005.docx]

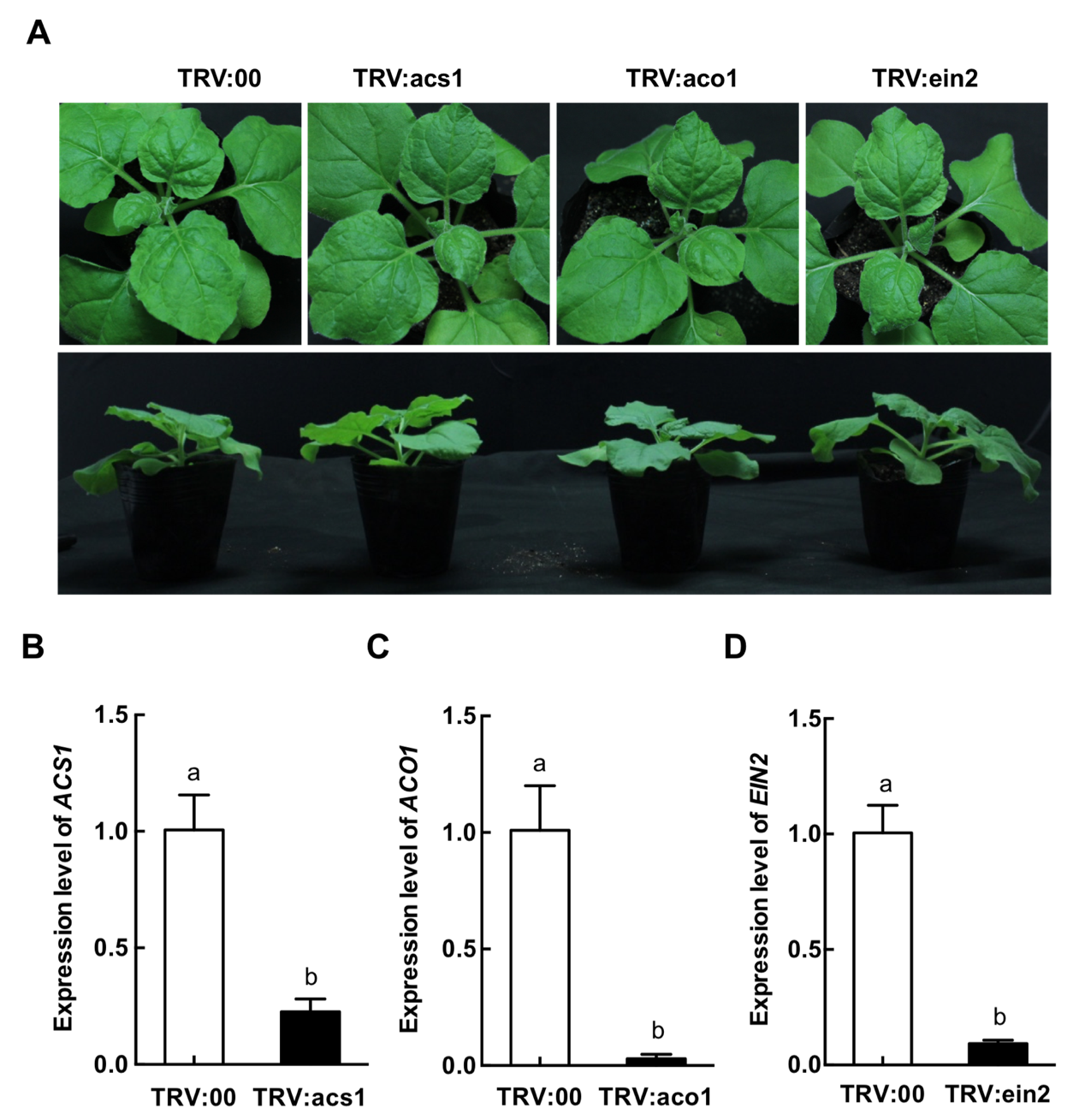


**Fig. S5 Silencing of *ACS1*, *ACO1* and *EIN2* in *N. benthamiana***

A. The phenotype of TRV:acs1, TRV:aco1 and TRV:ein2-treated *N. benthamiana* at 8 dpi. B-D. The expression of *ACS1, ACO1* and *EIN2* in plants was determined by quantitative RT-PCR. Results indicate the silencing of these genes in treated plants. Error bars represent the mean ± SD of three independent biological replicates. Different letters on histograms indicate significant differences (*p <* 0.05).
