# Supplementary material for: NbALD1 mediates resistance to turnip mosaic virus by regulating the accumulation of salicylic acid and the ethylene pathway in Nicotiana benthamiana
Source: Mol Plant Pathol. 2019 Apr 23;20(7):990–1004. doi: 10.1111/mpp.12808 (PMC6589722; doi:10.1111/mpp.12808)
Supplement: Supplementary file 7 — Fig. S7 ACC treatment induced the expression of ERF3. [file MPP-20-990-s007.docx]

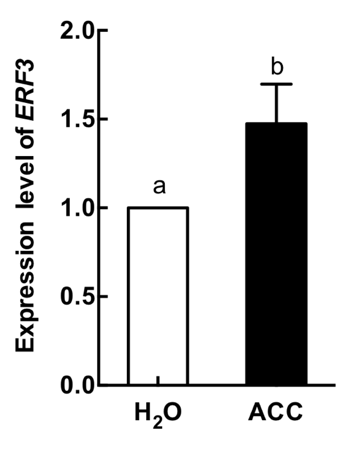


**Fig. S7 ACC treatment induced the expression of ERF3**

The expression levels of *ERF3* in H2O- and 20 μM ACC-treated plants as determined by qRT-PCR. Error bars represent the mean ± SD of three independent biological replicates. Different letters on histograms indicate significant differences (*p <* 0.05).
