# Supplementary material for: NbALD1 mediates resistance to turnip mosaic virus by regulating the accumulation of salicylic acid and the ethylene pathway in Nicotiana benthamiana
Source: Mol Plant Pathol. 2019 Apr 23;20(7):990–1004. doi: 10.1111/mpp.12808 (PMC6589722; doi:10.1111/mpp.12808)
Supplement: Supplementary file 8 — Fig. S8 Silencing of NbALD1 and ACS1 in N. benthamiana simultaneously. [file MPP-20-990-s008.docx]

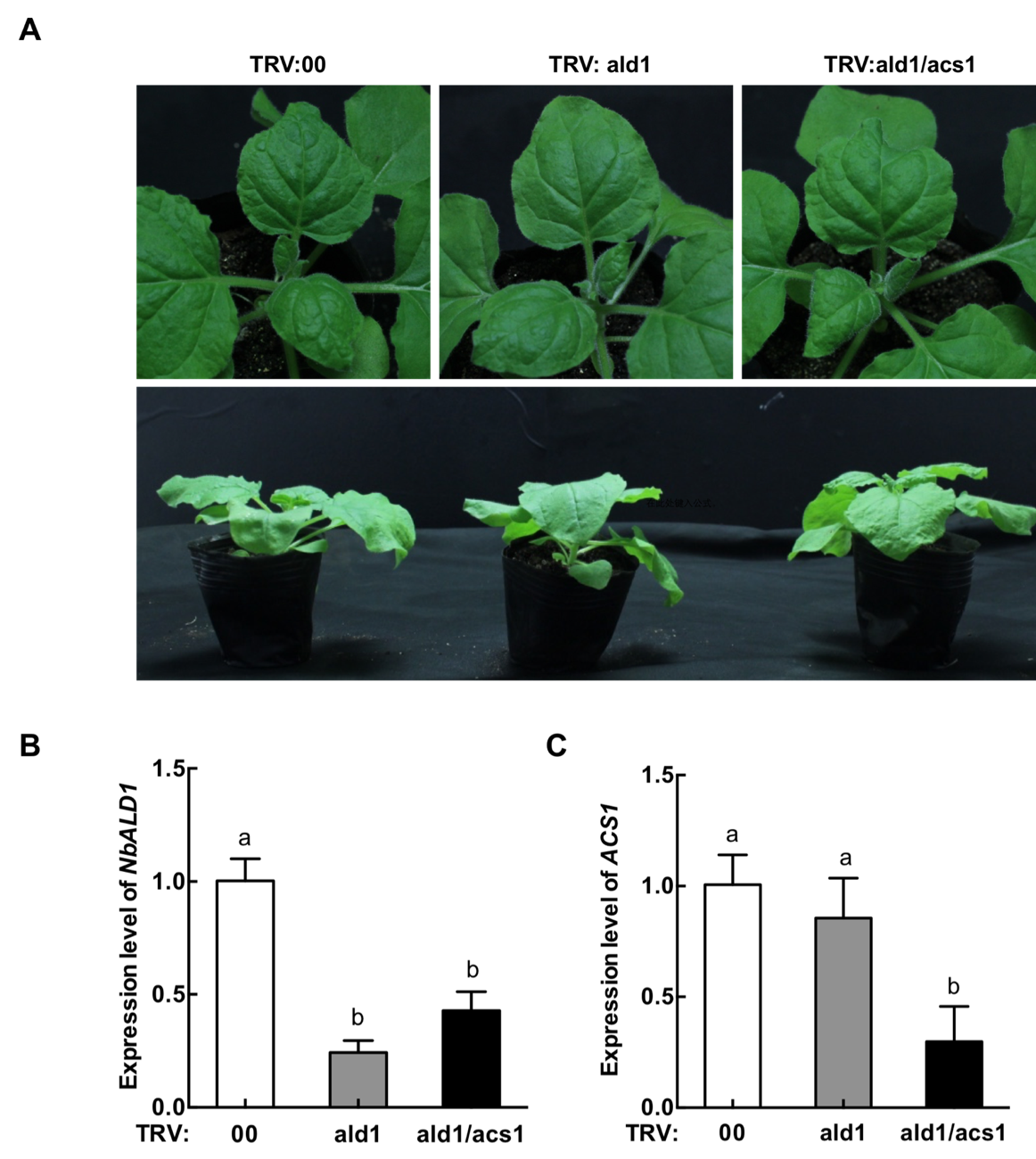


**Fig. S8 Silencing of *NbALD1* and *ACS1* in *N. benthamiana* simultaneously**

A. The phenotype of TRV:00, TRV:ald1 and TRV:ald1&acs1-treated *N. benthamiana* at 8 dpi. B. Expression levels of *ALD1* and *ACS1* in TRV:00, TRV:ald1 and TRV:ald1&acs1-treated *N. benthamiana*. Error bars represent the mean ± SD of three independent biological replicates. Different letters on histograms indicate significant differences (*p <* 0.05).
