# Supplementary material for: NbALD1 mediates resistance to turnip mosaic virus by regulating the accumulation of salicylic acid and the ethylene pathway in Nicotiana benthamiana
Source: Mol Plant Pathol. 2019 Apr 23;20(7):990–1004. doi: 10.1111/mpp.12808 (PMC6589722; doi:10.1111/mpp.12808)
Supplement: Supplementary file 9 — Fig. S9 Expression of ERF3 in TuMV‐infected wild‐type plants (A), NbALD1‐overexpressed plants (B) and expression of NbALD1 in TuMV‐infected ACS1‐silenced plants (C). [file MPP-20-990-s009.docx]

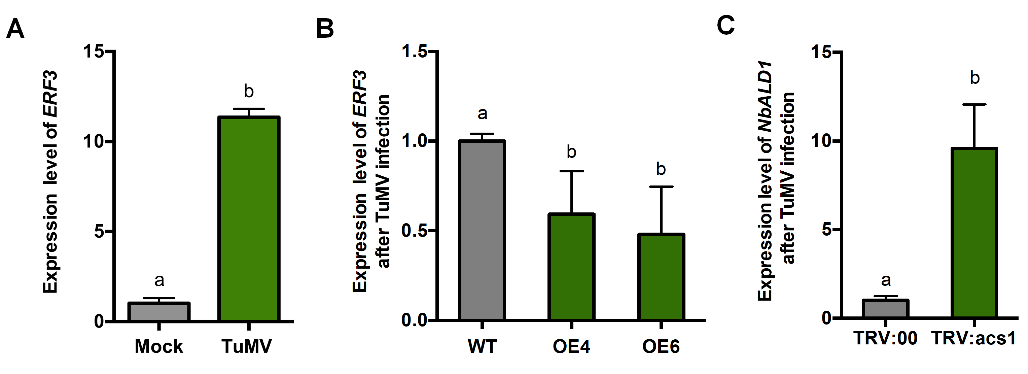


**Fig. S9** **Expression of *ERF3* in TuMV-infected wild type plants (A), *NbALD1*-overexpressed plants (B) and *ACS*-silenced plants (C).**
