# Supplementary material for: NbALD1 mediates resistance to turnip mosaic virus by regulating the accumulation of salicylic acid and the ethylene pathway in Nicotiana benthamiana
Source: Mol Plant Pathol. 2019 Apr 23;20(7):990–1004. doi: 10.1111/mpp.12808 (PMC6589722; doi:10.1111/mpp.12808)
Supplement: Supplementary file 10 — Table S1 Numbers of infection loci and statistical analysis. [file MPP-20-990-s010.docx]

Table 1A. The number of infection loci on mock and ALD1-silenced plants

| Plant number | TRV:00 | TRV:ald1 |
| --- | --- | --- |
| 1 | 15 | 33 |
| 2 | 21 | 42 |
| 3 | 4 | 48 |
| 4 | 14 | 38 |
| 5 | 7 | 45 |
| 6 | 17 | 47 |
| 7 | 15 | 51 |
| 8 | 13 | 49 |
| 9 | 8 | 37 |
| 10 | 23 | 36 |
| 11 | 27 | 29 |
| 12 | 13 | 43 |
| 13 | 10 | 49 |
| 14 | 22 | 38 |
| 15 | 20 | 35 |
| 16 | 6 | 49 |
| 17 | 25 | 50 |
| 18 | 6 | 44 |
| 19 | 17 | 30 |
| 20 | 19 | 39 |

| P value | < 0.0001 |
| --- | --- |
| P value summary | ** |
| Significantly different? (P < 0.05) | Yes |
| One- or two-tailed P value? | Two-tailed |
|  |  |

Table 1B. The number of infection loci on WT，OE4 and OE6 plants after TuMV infection

| Plant number | WT | OE#4 | OE#6 |
| --- | --- | --- | --- |
| 1 | 65 | 15 | 8 |
| 2 | 56 | 22 | 5 |
| 3 | 44 | 8 | 4 |
| 4 | 30 | 9 | 7 |
| 5 | 58 | 13 | 11 |
| 6 | 60 | 11 | 12 |
| 7 | 47 | 14 | 9 |
| 8 | 51 | 6 | 7 |
| 9 | 55 | 16 | 4 |
| 10 | 48 | 9 | 13 |
| 11 | 37 | 9 | 15 |
| 12 | 39 | 8 | 10 |
| 13 | 42 | 10 | 9 |
| 14 | 65 | 13 | 6 |
| 15 | 43 | 12 | 8 |
| 16 | 51 | 7 | 7 |
| 17 | 38 | 19 | 8 |
| 18 | 40 | 11 | 5 |
| 19 | 56 | 15 | 17 |
| 20 | 45 | 5 | 11 |

| OE#4 vs. WT | ** |  |  |  |  |  |
| --- | --- | --- | --- | --- | --- | --- |
| OE#6 vs. WT | ** |  |  |  |  |  |

Table 1C The number of infection loci on mock and ALD1-silenced plants that were pretreated with H_2_O and 100 μM Pip after TuMV infection

| number | TRV:ald1 | |
| --- | --- | --- |
|  | H_2_O | Pip |
| 1 | 78 | 68 |
| 2 | 91 | 48 |
| 3 | 55 | 40 |
| 4 | 56 | 35 |
| 5 | 48 | 22 |
| 6 | 76 | 30 |
| 7 | 82 | 21 |
| 8 | 61 | 17 |
| 9 | 64 | 23 |
| 10 | 62 | 37 |
| 11 | 50 | 19 |
| 12 | 73 | 41 |
| 13 | 44 | 32 |
| 14 | 62 | 14 |
| 15 | 57 | 19 |
| 16 | 55 | 22 |
| 17 | 65 | 33 |
| 18 | 49 | 27 |
| 19 | 70 | 20 |
| 20 | 53 | 37 |

P value summary: ** significant different? (P<0.05): Yes

Table 1D The number of infection loci on mock plants that were pretreated with H_2_O and 100 μM Pip after TuMV infection

| number | TRV:00 | |
| --- | --- | --- |
|  | H_2_O | Pip |
| 1 | 17 | 9 |
| 2 | 21 | 10 |
| 3 | 35 | 9 |
| 4 | 23 | 4 |
| 5 | 19 | 16 |
| 6 | 38 | 8 |
| 7 | 24 | 3 |
| 8 | 34 | 6 |
| 9 | 22 | 6 |
| 10 | 20 | 7 |
| 11 | 18 | 15 |
| 12 | 18 | 16 |
| 13 | 21 | 8 |
| 14 | 18 | 6 |
| 15 | 26 | 4 |
| 16 | 27 | 17 |
| 17 | 19 | 11 |
| 18 | 26 | 5 |
| 19 | 23 | 10 |
| 20 | 30 | 7 |

P value summary: ** significant different? (P<0.05): Yes

Table 1E. the number of infection loci on WT and *NahG* infected with TuMV.

| Plants number | WT | *NahG* |
| --- | --- | --- |
| 1 | 7 | 40 |
| 2 | 12 | 38 |
| 3 | 21 | 29 |
| 4 | 17 | 44 |
| 5 | 8 | 52 |
| 6 | 11 | 37 |
| 7 | 14 | 43 |
| 8 | 19 | 34 |
| 9 | 13 | 36 |
| 10 | 10 | 33 |
| 11 | 16 | 28 |
| 12 | 13 | 41 |
| 13 | 25 | 45 |
| 14 | 21 | 48 |
| 15 | 18 | 22 |
| 16 | 16 | 47 |
| 17 | 11 | 36 |
| 18 | 10 | 39 |
| 19 | 9 | 40 |
| 20 | 23 | 37 |
| P value summary | ** |  |
| Significantly different? (P < 0.05) | Yes |  |

Table 1F The number of infection loci on *N. benthamiana* plants that were pretreated with H_2_O and 10 μM SA after TuMV infection

| Number | H_2_O | 10 μM SA |
| --- | --- | --- |
| 1 | 233 | 20 |
| 2 | 168 | 90 |
| 3 | 222 | 29 |
| 4 | 173 | 71 |
| 5 | 199 | 65 |
| 6 | 245 | 55 |
| 7 | 179 | 105 |
| 8 | 214 | 78 |
| 9 | 182 | 96 |
| 10 | 203 | 91 |
| 11 | 197 | 37 |
| 12 | 234 | 62 |
| 13 | 175 | 68 |
| 14 | 219 | 43 |
| 15 | 186 | 49 |
| 16 | 177 | 51 |
| 17 | 215 | 88 |
| 18 | 169 | 69 |
| 19 | 174 | 73 |
| 20 | 216 | 67 |

| P value summary | ** |
| --- | --- |
| Significantly different? (P < 0.05) | Yes |

Table 1G. The number of infection loci on WT and *NahG* plants that pretreated with water and 100 pM Pip after TuMV infection

| Number | WT | | *NahG* | |
| --- | --- | --- | --- | --- |
|  | H_2_O | Pip | H_2_O | Pip |
| 1 | 34 | 4 | 29 | 18 |
| 2 | 19 | 9 | 55 | 17 |
| 3 | 29 | 4 | 46 | 28 |
| 4 | 26 | 6 | 43 | 26 |
| 5 | 14 | 9 | 48 | 23 |
| 6 | 33 | 8 | 36 | 21 |
| 7 | 25 | 8 | 51 | 20 |
| 8 | 35 | 7 | 63 | 27 |
| 9 | 17 | 11 | 54 | 32 |
| 10 | 31 | 3 | 42 | 36 |
| 11 | 28 | 5 | 47 | 22 |
| 12 | 30 | 10 | 56 | 15 |
| 13 | 23 | 9 | 52 | 34 |
| 14 | 27 | 7 | 39 | 33 |
| 15 | 24 | 7 | 41 | 29 |
| 16 | 27 | 13 | 45 | 29 |
| 17 | 19 | 11 | 38 | 24 |
| 18 | 21 | 19 | 28 | 26 |
| 19 | 37 | 4 | 43 | 31 |
| 20 | 15 | 6 | 38 | 16 |

|  |  |  |  |  |  |  |  |
| --- | --- | --- | --- | --- | --- | --- | --- |
| Pip/WT vs. H_2_O/WT | Yes | ** |  |  |  |  |  |
| H_2_O/nahG vs. H_2_O/WT | Yes | ** |  |  |  |  |  |
| Pip/nahG vs. H_2_O/WT | No | ns |  |  |  |  |  |
| H_2_O/nahG vs. Pip/WT | Yes | ** |  |  |  |  |  |
| Pip/nahG vs. Pip/WT | Yes | ** |  |  |  |  |  |
| Pip/nahG vs. H_2_O/nahG | Yes | ** |  |  |  |  |  |

Table 1H. The number of infection loci on mock, ACS1, ACO1, Ein2-silenced plants after TuMV infection

| number | TRV:00 | TRV:acs1 | TRV:aco1 | TRV:ein2 |
| --- | --- | --- | --- | --- |
| 1 | 90 | 27 | 17 | 20 |
| 2 | 65 | 11 | 19 | 32 |
| 3 | 58 | 48 | 8 | 42 |
| 4 | 61 | 27 | 20 | 26 |
| 5 | 92 | 19 | 20 | 39 |
| 6 | 55 | 25 | 29 | 35 |
| 7 | 75 | 31 | 3 | 25 |
| 8 | 72 | 23 | 17 | 32 |
| 9 | 97 | 18 | 20 | 24 |
| 10 | 57 | 37 | 17 | 22 |
| 11 | 63 | 26 | 14 | 36 |
| 12 | 91 | 30 | 25 | 14 |
| 13 | 73 | 22 | 27 | 18 |
| 14 | 64 | 17 | 29 | 24 |
| 15 | 68 | 17 | 17 | 32 |
| 16 | 77 | 35 | 19 | 18 |
| 17 | 83 | 32 | 13 | 23 |
| 18 | 81 | 16 | 34 | 21 |
| 19 | 67 | 29 | 9 | 15 |
| 20 | 83 | 21 | 18 | 26 |

|  |  |  |  |  |  |  |  |
| --- | --- | --- | --- | --- | --- | --- | --- |
| TRV:acs1 vs. TRV:00 | Yes | ** |  |  |  |  |  |
| TRV:aco1 vs. TRV:00 | Yes | ** |  |  |  |  |  |
| TRV:ein2 vs. TRV:00 | Yes | ** |  |  |  |  |  |
| TRV:aco1 vs. TRV:acs1 | No | ns |  |  |  |  |  |
| TRV:ein2 vs. TRV:acs1 | No | ns |  |  |  |  |  |
| TRV:ein2 vs. TRV:aco1 | Yes | * |  |  |  |  |  |

Table 1I. The number of infection loci on *N. benthamiana* that were pretreated with H_2_O and 10 μM AVG after TuMV infection

| Number | H_2_O | 10 μM AVG |
| --- | --- | --- |
| 1 | 116 | 28 |
| 2 | 89 | 65 |
| 3 | 86 | 12 |
| 4 | 187 | 24 |
| 5 | 87 | 37 |
| 6 | 113 | 33 |
| 7 | 122 | 47 |
| 8 | 139 | 51 |
| 9 | 84 | 26 |
| 10 | 76 | 39 |
| 11 | 90 | 18 |
| 12 | 155 | 58 |
| 13 | 161 | 62 |
| 14 | 115 | 67 |
| 15 | 106 | 35 |
| 16 | 94 | 27 |
| 17 | 103 | 31 |
| 18 | 99 | 25 |
| 19 | 126 | 16 |
| 20 | 111 | 34 |

| P value summary | ** |
| --- | --- |
| Significantly different? (P < 0.05) | Yes |

Table 1J. The number of infection loci on *N. benthamiana* that were pretreated with H_2_O and 100 μM ACC after TuMV infection

| Number | H_2_O | 100 μM ACC |
| --- | --- | --- |
| 1 | 10 | 103 |
| 2 | 41 | 78 |
| 3 | 34 | 87 |
| 4 | 17 | 157 |
| 5 | 11 | 94 |
| 6 | 22 | 104 |
| 7 | 38 | 115 |
| 8 | 27 | 96 |
| 9 | 42 | 77 |
| 10 | 15 | 69 |
| 11 | 24 | 132 |
| 12 | 56 | 147 |
| 13 | 64 | 88 |
| 14 | 33 | 97 |
| 15 | 25 | 106 |
| 16 | 53 | 83 |
| 17 | 47 | 85 |
| 18 | 41 | 104 |
| 19 | 23 | 117 |
| 20 | 37 | 96 |

| P value summary | ** |
| --- | --- |
| Significantly different? (P < 0.05) | Yes |

Table 1K. The number of infection loci on mock and ALD1-silenced and ALD1/ACS1-silenced plants after TuMV infection

| number | TRV:00 | TRV:ald1 | TRV:ald1/acs1 |
| --- | --- | --- | --- |
| 1 | 65 | 29 | 47 |
| 2 | 61 | 73 | 36 |
| 3 | 18 | 114 | 26 |
| 4 | 55 | 110 | 51 |
| 5 | 26 | 113 | 98 |
| 6 | 20 | 121 | 61 |
| 7 | 63 | 113 | 50 |
| 8 | 51 | 139 | 74 |
| 9 | 39 | 148 | 28 |
| 10 | 45 | 123 | 60 |
| 11 | 53 | 115 | 44 |
| 12 | 60 | 86 | 51 |
| 13 | 58 | 105 | 35 |
| 14 | 25 | 133 | 47 |
| 15 | 31 | 109 | 83 |
| 16 | 57 | 107 | 58 |
| 17 | 24 | 128 | 49 |
| 18 | 56 | 136 | 36 |
| 19 | 37 | 139 | 47 |
| 20 | 44 | 121 | 54 |

|  |  |  |  |  |  |  |  |
| --- | --- | --- | --- | --- | --- | --- | --- |
| TRV:ald1 vs. TRV:00 | Yes | ** |  |  |  |  |  |
| TRV:ald1/acs1 vs. TRV:00 | No | ns |  |  |  |  |  |
| TRV:ald1/acs1 vs. TRV:ald1 | Yes | ** |  |  |  |  |  |

Table 1L. The number of infection loci on water and Pip treated plants that sprayed with water and 100 μM ACC after TuMV infection

| Number | H_2_O | | 100 pM Pip | |
| --- | --- | --- | --- | --- |
|  | H_2_O | 100 μM ACC | H_2_O | 100 μM ACC |
| 1 | 56 | 244 | 18 | 66 |
| 2 | 57 | 116 | 14 | 96 |
| 3 | 66 | 164 | 35 | 29 |
| 4 | 77 | 118 | 1 | 95 |
| 5 | 86 | 98 | 2 | 45 |
| 6 | 117 | 155 | 48 | 79 |
| 7 | 77 | 150 | 43 | 53 |
| 8 | 58 | 223 | 23 | 26 |
| 9 | 65 | 145 | 22 | 34 |
| 10 | 62 | 178 | 17 | 33 |
| 11 | 74 | 136 | 31 | 105 |
| 12 | 81 | 119 | 4 | 101 |
| 13 | 103 | 151 | 2 | 65 |
| 14 | 76 | 162 | 41 | 47 |
| 15 | 64 | 187 | 33 | 86 |
| 16 | 60 | 159 | 27 | 96 |
| 17 | 56 | 136 | 38 | 27 |
| 18 | 72 | 142 | 19 | 99 |
| 19 | 79 | 133 | 27 | 35 |
| 20 | 108 | 177 | 25 | 55 |

|  |  | |  | | | | |  | |  |  |  |  |  |
| --- | --- | --- | --- | --- | --- | --- | --- | --- | --- | --- | --- | --- | --- | --- |
| ACC/ H_2_O vs. H_2_O/ H_2_O | Yes | ** | |  |  |  |  | |  |  |  |  |  |  |
| H_2_O/Pip vs. H_2_O/ H_2_O | Yes | ** | |  |  |  |  | |  |  |  |  |  |  |
| ACC/Pip vs. H_2_O/ H_2_O | No | ns | |  |  |  |  | |  |  |  |  |  |  |
| H_2_O/Pip vs. ACC/ H_2_O | Yes | ** | |  |  |  |  | |  |  |  |  |  |  |
| ACC/Pip vs. ACC/ H_2_O | Yes | ** | |  |  |  |  | |  |  |  |  |  |  |
| ACC/Pip vs. H_2_O/Pip | Yes | ** | |  |  |  |  | |  |  |  |  |  |  |

Table 1M The number of infection loci on mock and ald1-silenced plants that sprayed with water and 10 μM SA after TuMV infection

| Number | H_2_O | | 10 μM SA | |
| --- | --- | --- | --- | --- |
|  | TRV:00 | TRV:ald1 | TRV:00 | TRV:ald1 |
| 1 | 10 | 38 | 5 | 18 |
| 2 | 15 | 62 | 3 | 19 |
| 3 | 34 | 43 | 12 | 18 |
| 4 | 24 | 38 | 7 | 22 |
| 5 | 22 | 36 | 1 | 26 |
| 6 | 18 | 57 | 6 | 33 |
| 7 | 16 | 40 | 4 | 27 |
| 8 | 14 | 41 | 16 | 19 |
| 9 | 33 | 59 | 9 | 21 |
| 10 | 15 | 38 | 3 | 17 |
| 11 | 21 | 55 | 4 | 23 |
| 12 | 20 | 44 | 8 | 15 |
| 13 | 15 | 61 | 11 | 30 |
| 14 | 27 | 35 | 10 | 29 |
| 15 | 19 | 48 | 6 | 17 |
| 16 | 17 | 52 | 15 | 20 |
| 17 | 14 | 49 | 14 | 36 |
| 18 | 31 | 74 | 21 | 18 |
| 19 | 16 | 46 | 5 | 28 |
| 20 | 25 | 39 | 6 | 20 |

TRV:00/H_2_O vs TRV:ald1/H_2_O Yes **

TRV:00/H_2_O vs TRV:00/SA Yes **

TRV:00/H_2_O vs TRV:ald1/SA No ns

TRV:ald1/H_2_O vs TRV:00/SA Yes **

TRV:ald1/H_2_O vs TRV:ald1/SA Yes **

TRV:00/SA vs TRV:ald1/SA Yes **
