# Supplementary material for: NbALD1 mediates resistance to turnip mosaic virus by regulating the accumulation of salicylic acid and the ethylene pathway in Nicotiana benthamiana
Source: Mol Plant Pathol. 2019 Apr 23;20(7):990–1004. doi: 10.1111/mpp.12808 (PMC6589722; doi:10.1111/mpp.12808)
Supplement: Supplementary file 11 — Table S2 Primers used for analysis. [file MPP-20-990-s011.docx]

**Table S2. Primers for RNA Gel Blot Probes, Real-time PCR, and Semi-RT-PCR Related to Experimental Procedures**

| name | sequence | use |
| --- | --- | --- |
| TuCP(probe)-F | GGTTTGAAGGTGTAATGGCTGA | Northern blot probe |
| TuCP(probe)-R | ATATCGTGGCATGTATGGTCG |  |
| ALD1-F | TCCATATCCACTTCGCTTTTATT | Real qRT-PCR and Semi-RT-PCR |
| ALD1-R | TATTGCTTTGGCATTTGGGTA |  |
| ACO1-det-F | cggcctacaactcctcaaag |  |
| ACO1-det-R | agccaatgacattcgagtcc |  |
| ACS1-det-F | AATGCGGGACTTTTCTGTTG |  |
| ACS1-det-R | CCTCCCTAACGCAATATCCA |  |
| Ein2-det-F | TGCTGTTTTGTTTCCACTGC |  |
| EIn2-det-R | TGCAAATGCACTCTCTCCAC |  |
| FMO1-det-F | CCAGAAGTGTTCAAGGGTAAAGTTC |  |
| FMO1-det-R | CAGCACATTCAACAGCAAGATCTAT |  |
| ICS1-det-F | TTGTTGTCTTCACCTATGCGTTTAC |  |
| ICS1-det-R | AGAAAAGGTATGCACTTTAACACCG |  |
| ERF3-det-F | TACCGACGGAGAATCAGAGC |  |
| ERF3-det-R | CGCCACAAAGAATCAAAGAG |  |
| OE-F | CTGCCCGGGGCCTGGGGTACCATGTTTTCCATATCCACTTCGCTTT | Overexpression of NbALD1 |
| OE-R | CGATCGGGGAAATTCGAGCTCttaAGCGTAGTCTGGGACGTCGTATGGGTAACAAAGCAAGGTTGTTAG |  |
| VIGS-F | CgACgACAAgACCgTATGTTTTCCATATCCACTTCGC | Suppression of NbALD1 |
| VIGS-R | gAggAgAagAgCCgTCTACAATTTCTCTTCTTAGTTCTTTc |  |
| VIGS-NbACS1-F | CGACGACAAGACCGTGATGCATTTTTAGGATGCATTTTT | Suppression of ethylene signal pathway |
| VIGS-NbACS1-R | GAGGAGAAGAGCCGCCATGTCTTTGAAAAAGACTGT |  |
| VIGS-NbACO1-F | CGACGACAAGACCGTCGCCAGCGCCACTCCATCGTCG |  |
| VIGS-NbACO1-R | GAGGAGAAGAGCCGCTTTAAACCTCTTGACTACATATGT |  |
| VIGS-NbEIN2-F | CGACGACAAGACCGTCAGGCTGGCTACACCATGTG |  |
| VIGS-NbEIN2-R | GAGGAGAAGAGCCGCTAACCACAGCATCAGACAAAGAG |  |
