# Supplementary material for: NbALD1 mediates resistance to turnip mosaic virus by regulating the accumulation of salicylic acid and the ethylene pathway in Nicotiana benthamiana
Source: Mol Plant Pathol. 2019 Apr 23;20(7):990–1004. doi: 10.1111/mpp.12808 (PMC6589722; doi:10.1111/mpp.12808)
Supplement: Supplementary file 12 — Table S3 Numbers of plants showing fluorescence under UV light in the systemic leaves at different times and statistical analysis of infection rates. [file MPP-20-990-s012.docx]

**Table S3**

**Table S3 A. The record of the number of plants showing fluorescence under UV light at time course for TRV:00, TRV:ald1 plants Related to Figure 1 and Materials and Methods.**

| Time(dpi) | TRV:00 | | | TRV:ald1 | | |
| --- | --- | --- | --- | --- | --- | --- |
|  | R1^*1^ | R2 | R3 | R1 | R2 | R3 |
| 3 | 3 | 6 | 4 | 8 | 10 | 11 |
| 3.5 | 8 | 8 | 7 | 15 | 14 | 15 |
| 4 | 13 | 14 | 13 | 19 | 19 | 17 |
| 4.5 | 18 | 17 | 16 | 20 | 19 | 19 |
| 5 | 20 | 20 | 18 | 20 | 20 | 20 |

For each repeat, 20 plants were inoculated with TuMV-GFP.

*1: R1 means biological Repeat 1.

**Statistical analysis of infection rate for TRV:00, TRV:ald1. Related to Figure 1 and Materials and Methods.**

|  | Time(h) | TRV:ald1 |
| --- | --- | --- |
| compared to TRV:00 | 3 | * |
|  | 3.5 | ** |
|  | 4 | ** |
|  | 4.5 | * |
|  | 5 | O |

“O” represents no significant difference, **P*<0.05, ** *P*<0.01.

**Table S3 B. The record of the number of plants showing fluorescence under UV light at time course for WT, OE4, and OE6 plants Related to Figure 2 and Materials and Methods**

| Time (dpi) | WT | | | OE4 | | | OE6 | | | |
| --- | --- | --- | --- | --- | --- | --- | --- | --- | --- | --- |
|  | R1 | R2 | R3 | R1 | R2 | R3 | R1 | R2 | R3 |  |
| 3.5 | 7 | 6 | 7 | 4 | 3 | 3 | 5 | 7 | 5 |  |
| 4 | 14 | 12 | 15 | 8 | 7 | 10 | 10 | 11 | 12 |  |
| 4.5 | 18 | 17 | 18 | 12 | 13 | 11 | 15 | 12 | 15 |  |
| 5 | 20 | 19 | 18 | 15 | 17 | 18 | 17 | 17 | 17 |  |
| 5.5 | 20 | 20 | 20 | 19 | 20 | 17 | 20 | 19 | 20 |  |

**Statistical analysis of infection rate for WT, OE4 OE6, Related to Figure 1 and Materials and Methods.**

|  | Time(h) | OE4 | OE6 |
| --- | --- | --- | --- |
| Compared to WT | 3.5 | ** | O |
|  | 4 | * | O |
|  | 4.5 | ** | * |
|  | 5 | O | * |
|  | 5.5 | O | O |

“O” represents no significant difference, **P*<0.05, ** *P*<0.01.

**Table S3 C. The record of the number of plants showing fluorescence under UV light at time course for H_2_O and 100 pM Pip pretreated mock plants Related to Figure 3 and Materials and Methods**

| Time(dpi) | Mock | | | | | |
| --- | --- | --- | --- | --- | --- | --- |
|  | H_2_O | | | Pip | | |
|  | R1 | R2 | R3 | R1 | R2 | R3 |
| 3 | 5 | 6 | 7 | 4 | 4 | 3 |
| 3.5 | 9 | 13 | 14 | 6 | 7 | 6 |
| 4 | 16 | 15 | 15 | 13 | 11 | 12 |
| 4.5 | 18 | 19 | 18 | 15 | 14 | 15 |
| 5 | 20 | 20 | 20 | 18 | 17 | 17 |
| 5.5 | 20 | 20 | 20 | 20 | 20 | 20 |

**Statistical analysis of infection rate for H_2_O and 100 pM Pip pretreated mock plants Related to Figure 3 and Materials and Methods.**

| Mock | Time(dpi) | 100 pM Pip |
| --- | --- | --- |
| Compare to H_2_O | 3.0 | * |
|  | 3.5 | * |
|  | 4 | * |
|  | 4.5 | ** |
|  | 5 | ** |
|  | 5.5 | O |

“O” represents no significant difference, **P*<0.05, ** *P*<0.01.

**Table S3 D. The record of the number of plants showing fluorescence under UV light at time course for H_2_O and 100 pM Pip pretreated *NbALD1*-silenced plants Related to Figure 3 and Materials and Methods**

| Time(dpi) | TRV:ald1 | | | | | |
| --- | --- | --- | --- | --- | --- | --- |
|  | H_2_O | | | Pip | | |
|  | R1 | R2 | R3 | R1 | R2 | R3 |
| 3 | 10 | 10 | 13 | 7 | 7 | 8 |
| 3.5 | 15 | 14 | 17 | 11 | 12 | 13 |
| 4 | 19 | 18 | 17 | 18 | 17 | 17 |
| 4.5 | 20 | 20 | 19 | 19 | 18 | 19 |
| 5 | 20 | 20 | 20 | 20 | 20 | 20 |

**Statistical analysis of infection rate for H_2_O and 100 pM Pip pretreated mock plants Related to Figure 3 and Materials and Methods.**

| TRV:00 | Time(dpi) | Pip |
| --- | --- | --- |
| Compare to H_2_O | 3 | * |
|  | 3.5 | * |
|  | 4 | O |
|  | 4.5 | O |
|  | 5 | O |

“O” represents no significant difference, **P*<0.05, ** *P*<0.01.

**Table S3 E. The record of the number of plants showing fluorescence under UV light at time course for WT and *NahG* plants Related to Figure 4 and Materials and Methods**

| Time(dpi) | WT | | | *NahG* | | |
| --- | --- | --- | --- | --- | --- | --- |
|  | R1 | R2 | R3 | R1 | R2 | R3 |
| 3 | 3 | 1 | 2 | 4 | 2 | 3 |
| 3.5 | 3 | 3 | 4 | 14 | 6 | 7 |
| 4 | 8 | 8 | 10 | 19 | 13 | 13 |
| 4.5 | 13 | 17 | 14 | 20 | 19 | 18 |
| 5 | 19 | 18 | 19 | 20 | 20 | 20 |
| 5.5 | 20 | 20 | 20 | 20 | 20 | 20 |

**Statistical analysis of infection rate for WT and *NahG* plants Related to Figure 4 and Materials and Methods.**

|  | Time(dpi) | *NahG* |
| --- | --- | --- |
| Compared to WT | 3 | O |
|  | 3.5 | O |
|  | 4 | * |
|  | 4.5 | * |
|  | 5 | * |
|  | 5.5 | O |

“O” represents no significant difference, **P*<0.05, ** *P*<0.01.

**Table S3 F. The record of the number of plants showing fluorescence under UV light at time course for H_2_O and 10 μM SA pretreated plants Related to Figure 4 and Materials and Methods**

| Time(dpi) | H_2_O | | | 10 μM SA | | |
| --- | --- | --- | --- | --- | --- | --- |
|  | R1 | R2 | R3 | R1 | R2 | R3 |
| 3 | 5 | 8 | 7 | 1 | 0 | 2 |
| 3.5 | 8 | 14 | 13 | 4 | 4 | 5 |
| 4 | 16 | 17 | 17 | 8 | 9 | 11 |
| 4.5 | 20 | 19 | 20 | 15 | 14 | 15 |
| 5 | 20 | 20 | 20 | 19 | 17 | 20 |

**Statistical analysis of infection rate for H_2_O and 10 μM SA pretreated plants Related to Figure 4 and Materials and Methods.**

|  | Time(dpi) | 10 μM SA |
| --- | --- | --- |
| Compared to H_2_O | 3 | ** |
|  | 3.5 | * |
|  | 4 | ** |
|  | 4.5 | ** |
|  | 5 | O |

“O” represents no significant difference, **P*<0.05, ** *P*<0.01.

**Table S3 G. The record of the number of plants showing fluorescence under UV light at time course for H_2_O and 100 pM Pip** **pretreated WT and *NahG* plants. Related to Figure 5 and Materials and Methods**

| Time  (dpi) | WT | | | | | | *NahG* | | | | | |
| --- | --- | --- | --- | --- | --- | --- | --- | --- | --- | --- | --- | --- |
|  | H_2_O | | | Pip | | | H_2_O | | | Pip | | |
|  | R1 | R2 | R3 | R1 | R2 | R3 | R1 | R2 | R3 | R1 | R2 | R3 |
| 3 | 5 | 2 | 2 | 1 | 0 | 0 | 4 | 2 | 3 | 3 | 1 | 2 |
| 3.5 | 8 | 3 | 4 | 4 | 2 | 2 | 14 | 6 | 7 | 7 | 5 | 5 |
| 4 | 13 | 11 | 10 | 8 | 7 | 7 | 19 | 13 | 13 | 14 | 10 | 11 |
| 4.5 | 18 | 17 | 14 | 11 | 15 | 13 | 20 | 19 | 18 | 18 | 17 | 16 |
| 5 | 19 | 18 | 19 | 15 | 17 | 15 | 20 | 20 | 20 | 20 | 18 | 18 |
| 5.5 | 20 | 20 | 20 | 19 | 18 | 18 | 20 | 20 | 20 | 20 | 20 | 20 |

**Statistical analysis of infection rate for H_2_O and 100 pM Pip** **pretreated WT and *NahG* plants. Related to Figure 5 and Materials and Methods.**

| Time  (dpi) |  |  | WT | | *NahG* | |
| --- | --- | --- | --- | --- | --- | --- |
|  |  |  | H_2_O | Pip | H_2_O | Pip |
| 3 | WT | H_2_O | # |  |  |  |
|  |  | Pip | O | # |  |  |
|  | *NahG* | H_2_O | O | * | # |  |
|  |  | Pip | O | O | O | # |
| 3.5 | WT | H_2_O | # |  |  |  |
|  |  | Pip | O | # |  |  |
|  | *NahG* | H_2_O | O | O | # |  |
|  |  | Pip | O | * | O | # |
| 4 | WT | H_2_O | # |  |  |  |
|  |  | Pip | * | # |  |  |
|  | *NahG* | H_2_O | O | * | # |  |
|  |  | Pip | O | * | O | # |
| 4.5 | WT | H_2_O | # |  |  |  |
|  |  | Pip | O | # |  |  |
|  | *NahG* | H_2_O | O | ** | # |  |
|  |  | Pip | O | * | O | # |
| 5 | WT | H_2_O | # |  |  |  |
|  |  | Pip | * | # |  |  |
|  | *NahG* | H_2_O | * | ** | # |  |
|  |  | Pip | O | * | O | # |
| 5.5 | WT | H_2_O | # |  |  |  |
|  |  | Pip | ** | # |  |  |
|  | *NahG* | H_2_O | O | ** | # |  |
|  |  | Pip | O | ** | O | # |

“O” represents no significant difference, **P*<0.05, ** *P*<0.01. # means the same treatment.

**Table S3 H. The record of the number of plants showing fluorescence under UV light at time course for mock, *ACS1*-, *ACO1*-, *Ein2*-silenced plants. Related to Figure 7 and Materials and Methods**

| Time  (dpi) | TRV:00 | | | TRV:acs1 | | | TRV:aco1 | | | TRV:ein2 | | |
| --- | --- | --- | --- | --- | --- | --- | --- | --- | --- | --- | --- | --- |
|  | R1 | R2 | R3 | R1 | R2 | R3 | R1 | R2 | R3 | R1 | R2 | R3 |
| 3 | 5 | 4 | 7 | 1 | 4 | 1 | 0 | 2 | 2 | 3 | 2 | 0 |
| 3.5 | 14 | 15 | 13 | 6 | 9 | 8 | 5 | 7 | 9 | 7 | 10 | 9 |
| 4 | 20 | 19 | 18 | 17 | 14 | 15 | 16 | 13 | 15 | 14 | 13 | 17 |
| 4.5 | 20 | 20 | 20 | 19 | 18 | 18 | 17 | 16 | 19 | 17 | 18 | 20 |
| 5 | 20 | 20 | 20 | 20 | 20 | 20 | 20 | 17 | 20 | 19 | 20 | 20 |

**Statistical analysis of infection rate for mock, *ACS1*-, *ACO1*-, *Ein2*-silenced plants. Related to Figure 7 and Materials and Methods.**

|  | Time(dpi) | TRV:acs1 | TRV:aco1 | TRV:ein2 |
| --- | --- | --- | --- | --- |
| Compared to TRV:00 | 3 | O | * | * |
|  | 3.5 | ** | ** | ** |
|  | 4 | * | * | * |
|  | 4.5 | ** | * | O |
|  | 5 | O | O | O |

“O” represents no significant difference, **P*<0.05, ** *P*<0.01.

**Table S3 I. The record of the number of plants showing fluorescence under UV light at time course for H_2_O and 100 μM ACC sprayed plants that were pretreated with H_2_O and 100 pM Pip. Related to Figure 8 and Materials and Methods**

| Time  (dpi) | H_2_O | | | | | | 100 pM Pip | | | | | |
| --- | --- | --- | --- | --- | --- | --- | --- | --- | --- | --- | --- | --- |
|  | H_2_O | | | ACC | | | H_2_O | | | ACC | | |
|  | R1 | R2 | R3 | R1 | R2 | R3 | R1 | R2 | R3 | R1 | R2 | R3 |
| 3.5 | 3 | 4 | 4 | 5 | 7 | 4 | 1 | 2 | 2 | 1 | 4 | 2 |
| 4 | 8 | 10 | 7 | 13 | 12 | 11 | 1 | 4 | 2 | 5 | 9 | 7 |
| 4.5 | 13 | 13 | 14 | 19 | 17 | 15 | 7 | 9 | 11 | 12 | 10 | 13 |
| 5 | 17 | 18 | 20 | 20 | 19 | 20 | 13 | 14 | 12 | 16 | 17 | 17 |
| 5.5 | 20 | 20 | 20 | 20 | 20 | 20 | 17 | 19 | 20 | 20 | 20 | 20 |

**Statistical analysis of infection rate for H_2_O and 100 μM ACC sprayed plants that were pretreated with H_2_O and 100 pM Pip. Related to Figure 8 and Materials and Methods.**

| Time  (dpi) |  |  | H_2_O | | Pip | |
| --- | --- | --- | --- | --- | --- | --- |
|  |  |  | H_2_O | ACC | H_2_O | ACC |
| 3.5 | H_2_O | H_2_O | # |  |  |  |
|  |  | ACC | O | # |  |  |
|  | Pip | H_2_O | * | * | # |  |
|  |  | ACC | O | O | O | # |
| 4 | H_2_O | H_2_O | # |  |  |  |
|  |  | ACC | * | # |  |  |
|  | Pip | H_2_O | ** | ** | # |  |
|  |  | ACC | O | * | * | # |
| 4.5 | H_2_O | H_2_O | # |  |  |  |
|  |  | ACC | * | # |  |  |
|  | Pip | H_2_O | * | ** | # |  |
|  |  | ACC | O | * | O | # |
| 5 | H_2_O | H_2_O | # |  |  |  |
|  |  | ACC | O | # |  |  |
|  | Pip | H_2_O | ** | ** | # |  |
|  |  | ACC | O | ** | ** | # |
| 5.5 | H_2_O | H_2_O | # |  |  |  |
|  |  | ACC | O | # |  |  |
|  | Pip | H_2_O | O | O | # |  |
|  |  | ACC | O | O | O | # |

“O” represents no significant difference, **P*<0.05, ** *P*<0.01. # means the same treatment.
